# Dimensionality of genomic information and its impact on genome-wide associations and variant selection for genomic prediction: a simulation study

**DOI:** 10.1186/s12711-023-00823-0

**Published:** 2023-07-17

**Authors:** Sungbong Jang, Shogo Tsuruta, Natalia Galoro Leite, Ignacy Misztal, Daniela Lourenco

**Affiliations:** Department of Animal and Dairy Science, University of Georgia, Athens, GA 30602 USA

## Abstract

**Background:**

Identifying true positive variants in genome-wide associations (GWA) depends on several factors, including the number of genotyped individuals. The limited dimensionality of genomic information may give insights into the optimal number of individuals to be used in GWA. This study investigated different discovery set sizes based on the number of largest eigenvalues explaining a certain proportion of variance in the genomic relationship matrix (**G**). In addition, we investigated the impact on the prediction accuracy by adding variants, which were selected based on different set sizes, to the regular single nucleotide polymorphism (SNP) chips used for genomic prediction.

**Methods:**

We simulated sequence data that included 500k SNPs with 200 or 2000 quantitative trait nucleotides (QTN). A regular 50k panel included one in every ten simulated SNPs. Effective population size (*Ne*) was set to 20 or 200. GWA were performed using a number of genotyped animals equivalent to the number of largest eigenvalues of **G** (EIG) explaining 50, 60, 70, 80, 90, 95, 98, and 99% of the variance. In addition, the largest discovery set consisted of 30k genotyped animals. Limited or extensive phenotypic information was mimicked by changing the trait heritability. Significant and large-effect size SNPs were added to the 50k panel and used for single-step genomic best linear unbiased prediction (ssGBLUP).

**Results:**

Using a number of genotyped animals corresponding to at least EIG98 allowed the identification of QTN with the largest effect sizes when *Ne* was large. Populations with smaller *Ne* required more than EIG98. Furthermore, including genotyped animals with a higher reliability (i.e., a higher trait heritability) improved the identification of the most informative QTN. Prediction accuracy was highest when the significant or the large-effect SNPs representing twice the number of simulated QTN were added to the 50k panel.

**Conclusions:**

Accurately identifying causative variants from sequence data depends on the effective population size and, therefore, on the dimensionality of genomic information. This dimensionality can help identify the most suitable sample size for GWA and could be considered for variant selection, especially when resources are restricted. Even when variants are accurately identified, their inclusion in prediction models has limited benefits.

**Supplementary Information:**

The online version contains supplementary material available at 10.1186/s12711-023-00823-0.

## Background

Several factors influence the statistical power required to identify causative variants in genome-wide associations (GWA), including the number of quantitative trait nucleotides (QTN) that affect a trait, the number of single nucleotide polymorphisms (SNPs) in the discovery panel, the number of genotyped individuals [1], and the size of the genomic blocks segregating in the population [2], among others. These genomic blocks are chromosome segments inherited from founders and are subject to recombination at each generation. Stam [3] showed that the size of these segments varies but with a mean size of 1/4*Ne*, where *Ne* is the effective population size. Given a species with a genome length equal to *L* Morgans, the number of independent chromosome segments (*Me*) segregating in a population can be calculated as 4*NeL*.

Animal populations have smaller *Ne* than human populations, which means smaller *Me*. Pocrnic et al. [4] showed that although millions of individuals can be genotyped, non-redundant information is finite, which means that genomic information has a limited dimensionality; therefore, the additive genetic information in a population is contained in a limited *Me*. The same authors related the limited dimensionality to *Me* = 4*NeL* and observed that this quantity corresponds to the number of largest eigenvalues (EIG) explaining 98% (EIG98) of the variance of the genomic relationship matrix (${\mathbf{G}}$). In cattle populations, EIG98 varies from 10 to 14K, and in pigs and chickens, it is about 4K. The minimum number of SNPs needed to cover those segments is approximately 12 *Me* [5].

With the availability of sequence information, causal variants are expected to be included in the data, which generates more opportunities for discovery than mid-density SNP panels [6]. When the causal variants are known and included in the standard SNP panels, the prediction accuracy of genomic estimated breeding values (GEBV) should increase. This is clearly observed in simulation studies where the QTN and their effects are known [7, 8]. However, the increase in accuracy by using significant variants from sequence data in real populations is almost zero [9–11]. This raises a question about the effectiveness of GWA in real populations. Although most traits of economic importance in farm animal populations are polygenic, in most cases, only a few peaks in GWA studies are statistically associated with traits of interest.

Misztal et al. [12] investigated the distribution of estimated SNP effects around the QTN and the ability to identify QTN depending on the *Ne* in simulated populations. They found that identifying QTN in populations with small *Ne* (i.e., 60) required three times more genotyped animals with phenotypes than in populations with large *Ne* (i.e., 600). However, not all simulated QTN were identified, regardless of the *Ne* or the amount of data. Distinguishing between noise and the true signal is more difficult in small populations because of longer chromosome segments and the uncertainty about the exact location of QTN. In addition, the level of noise may mask the signal and thus prevent the detection of associations. With sequence data, a high GWA resolution may be even harder to achieve for small populations due to the reasons mentioned above.

Although it is well known that increasing the sample size for GWA improves the resolution, to date, the links between the number of genotyped individuals, *Ne*, *Me*, and GWA resolution are unknown. Understanding the appropriate sample size for variant discovery, especially with sequence data, can help alleviate the economic and computational costs for practical applications. In addition, it could help manage resources when working with traits that are difficult and costly to record. Based on the limited dimensionality of genomic information, there may be an optimal number of animals that carry all the independent chromosome segments segregating in the population, and consequently, all the genomic information available in the population [4]. When animals have lots of information (i.e., own records or progeny records), GEBV are estimated with high accuracy. Knowing that SNP effects can be back-solved from GEBV [13] raises the question of whether the GWA resolution is high when *Me* animals with high reliability GEBV are used. Therefore, we hypothesize that the ability to identify causative variants is high when animals with high-reliability GEBV are used, and the sample size for GWA approaches *Me.* Thus, using a larger sample size may not improve further the GWA resolution or increase the proportion of variance explained by significant QTN. Here, we used the number of eigenvalues explaining different proportions of the variance in ${\mathbf{G}}$ to assess the dimensionality of genomic information and applied this number as the sample size in GWA. This allowed us to investigate the GWA resolution and the proportion of variance explained by significant QTN, given the dimensionality. We used simulated populations with varying *Ne*, varying numbers of QTN, and varying amounts of information for genotyped individuals. We also evaluated the impact of incorporating pre-selected variants, from GWA with different sample sizes based on dimensionality, to a 50k SNP chip for genomic prediction using single-step genomic best linear unbiased prediction (ssGBLUP).

## Methods

### Data simulation

To mimic the bovine genome, we simulated 29 chromosomes with a total length of 23.19 Morgan using the QMSim software [14]. All simulation parameter files are provided in Additional files 1, 2, 3, 4, 5, 6, 7, 8, 9, 10, 11, and 12. The overall number of SNPs was 500,000, all with a minor allele frequency higher than 0.05, whereas different numbers of QTN, i.e., 200 and 2000, were used for scenarios Q200 and Q2000, respectively, all with a minor allele frequency higher than 0.05. Biallelic SNPs and QTN were randomly placed on each chromosome, with the number of SNPs ranging from 9000 to 35,000 and that of QTN from 8 to 31 (Q200) or from 80 to 320 (Q2000). The QTN effects were sampled from a gamma distribution with a shape parameter of 0.4 and a scale parameter calculated internally for a genetic variance of 0.3, 0.9, and 0.99, depending on the scenario. It was assumed that simulated QTN explained all the additive genetic variance. A recurrent mutation rate of $2.5 \times 10^{ - 5}$ was assumed for both the SNPs and QTN. A regular 50k panel was created for genomic predictions, including one in every ten simulated SNPs.

A quantitative trait was simulated with a heritability of 0.3, 0.9, or 0.99 by setting the additive genetic variance to 0.3, 0.9, or 0.99, respectively, and keeping the phenotypic variance constant at 1.0. The different heritabilities mimicked limited (animals with low-reliability GEBV) or extensive (animals with high-reliability GEBV) phenotypic information [15]. The historical population was simulated for 2000 non-overlapping generations with an increase in size from 1000 (generation − 2000) to 50,000 (generation − 1000), and a decrease from 50,000 (generation − 999) to 20,000 (generation 0) to create linkage disequilibrium (LD) and mutation–drift equilibrium. Random mating and no selection or migration were assumed in the historical population. Recent populations with an *Ne* equal to 20 (*Ne*20) or 200 (*Ne*200) were simulated by changing the number of breeding males from 5 to 50 but keeping the number of females at 15,000. The founders of the recent populations came from generation 0 of the historical population. Twenty generations of random mating were carried out, considering a replacement rate of 80% for sires and 30% for dams. Animals were randomly selected and culled based on age. In total, 315,005 and 315,050 animals were generated in the recent population for *Ne*20 and *Ne*200, respectively. However, only animals from generations 11 to 20 had phenotypic and pedigree information that was used for the current study. Of those, 75,000 animals from generations 16 to 20 were genotyped (N = 15,000 in each generation). Each phenotype was the sum of an overall mean equal to 1.0, the true breeding value (TBV), and a random residual effect. Each simulated scenario represented a different combination of *Ne*, number of QTN, and heritability, and was replicated five times.

### Genotype scenarios—different heritabilities and sequence data

Limited or extensive phenotypic information for simulated animals was mimicked by changing the trait heritability. Based on a single record per animal, the reliability of the EBV equals the heritability [16]. If animals have a large number of progeny records, the reliability of their EBV would be higher. Therefore, the scenarios for which the heritabilities of the trait were 0.3, 0.9, and 0.99 represented simulations with animals that have breeding values with a low reliability (H30), high reliability (H90), and very high reliability (H99), respectively. Consequently, the higher the heritability, the greater the amount of information on the simulated animals without directly changing the number of records assigned to them [15]. Furthermore, after these simulations, sequence data scenarios were created. According to the general assumption for sequence data, we assumed that the QTN were contained in the genotypic data; therefore, the 500k SNPs and QTN were combined based on the map files in a post-processing step because the QMSim software simulates SNPs and QTN, separately. The descriptions of all the scenarios and combinations used are in Table 1.

**Table 1:** Description of all GWA scenarios

Scenario description	*Ne*	Number of QTN	Heritability
*Ne*20 Q200 H30	20	200	0.3
*Ne*20 Q200 H90	20	200	0.9
*Ne*20 Q200 H99	20	200	0.99
*Ne*20 Q2000 H30	20	2000	0.3
*Ne*20 Q2000 H90	20	2000	0.9
*Ne*20 Q2000 H99	20	2000	0.99
*Ne*200 Q200 H30	200	200	0.3
*Ne*200 Q200 H90	200	200	0.9
*Ne*200 Q200 H99	200	200	0.99
*Ne*200 Q2000 H30	200	2000	0.3
*Ne*200 Q2000 H90	200	2000	0.9
*Ne*200 Q2000 H99	200	2000	0.99

### Discovery, training, and test sets

Before the GWA analyses, all genotyped animals were separated into three non-overlapping datasets: discovery, training, and test. The test set was composed of genotyped animals from the last generation (N = 15,000), and the remaining genotyped animals (N = 60,000) were randomly assigned to the discovery and training sets (N = 30,000, respectively). The discovery sets were generated for GWA, and the training and test sets for genomic prediction. To test the possible bias in genomic prediction due to using the same dataset for discovery and training, two different schemes were designed: (1) discovery = training: genotyped animals used for discovery were also used for training, and (2) discovery $\ne$ training: different sets of genotyped animals were used for discovery and training.

### EIGx scenarios for discovery and training

Different scenarios based on the dimensionality of genomic information were used for discovery (GWA) and training (genomic prediction) to explore the impact of varying sample sizes on GWA and genomic prediction. The number of genotyped animals in each discovery and training set (EIG*x*) was equivalent to the number of largest eigenvalues explaining *x* percent of the variance in ${\mathbf{G}}$, where *x* was assumed to have the values 50, 60, 70, 80, 90, 95, 98, or 99. For example, the average number of largest eigenvalues explaining 50% of the variance in ${\mathbf{G}}$ was 521.6 ± 7.9 in the *Ne*200 Q2000 H30 scenario (Table 2) from five replicates. An additional scenario (ALL) in which the discovery and training sets included all available genotyped animals (N = 30,000) was also evaluated. The number of largest eigenvalues explaining *x* percent (50, 60, 70, 80, 90, 95, 98, 99) of the variance in ${\mathbf{G}}$ was computed using the preGSf90 program [17], which in a nutshell, computes the singular value decomposition of the matrix of genotypes centered for current allele frequencies (${\mathbf{M}}$) and squares the singular values to obtain eigenvalues. Then, the eigenvalues are sorted from the largest to the smallest and the largest eigenvalues are summed up to the desired *x* percent. All the simulated genotyped animals (N = 75,000) were used in the computations, and ${\mathbf{M}}$ contained 500k SNPs (but no QTN) centered for current allele frequencies. All genotyped animals for each discovery and training set were randomly selected beginning from the scenario explaining the lowest proportion of variance (EIG50). To ensure consistent results, we kept all the animals from the previous scenario in the next one, e.g., genotyped animals in EIG60 contained all those from EIG50. The numbers of genotyped animals used as discovery and training sets in each scenario are in Table 2.

**Table 2:** Number of genotyped animals (mean ± SE) for all scenarios in discovery and training sets of five replicates with an effective population size (*Ne*) of 20 or 200

*Ne*		Q200 H30	Q200 H90	Q200 H99	Q2000 H30	Q2000 H90	Q2000 H99
20	EIG50	75.4 ± 1.4	75.2 ± 1.8	76.6 ± 1.1	73.6 ± 1.7	75.4 ± 0.9	73.6 ± 0.9
	EIG60	128.6 ± 2.2	129.2 ± 2.9	130.2 ± 1.8	126 ± 3.2	128 ± 1.6	125.6 ± 1.7
	EIG70	216.4 ± 3.2	219.4 ± 4.1	220.2 ± 3.1	212.4 ± 4.9	215.6 ± 3.5	211.4 ± 2.4
	EIG80	386.8 ± 4.5	391.4 ± 6.3	394.6 ± 5.3	381.4 ± 7.8	383.4 ± 5.1	376.4 ± 4.7
	EIG90	856 ± 9.7	866.6 ± 14.1	871 ± 10.2	844.2 ± 13.9	844.6 ± 13.3	832.8 ± 11.7
	EIG95	1699.6 ± 24.1	1719 ± 26.2	1728 ± 18.7	1684.2 ± 25.2	1673.6 ± 26	1657.8 ± 24.4
	EIG98	3917.6 ± 52.6	3952 ± 43.0	3967.4 ± 37.6	3896.2 ± 47.8	3853.8 ± 50.8	3838.4 ± 55.1
	EIG99	6884.4 ± 75.7	6932.2 ± 53.6	6950.2 ± 54.5	6859 ± 64.2	6780 ± 69.4	6773.6 ± 83.8
	All	30,000	30,000	30,000	30,000	30,000	30,000
200	EIG50	512.6 ± 4.8	516.8 ± 3.5	514.0 ± 7.0	521.6 ± 7.9	513.0 ± 9.5	518.2 ± 5.0
	EIG60	892.4 ± 8.1	899.6 ± 5.6	895.8 ± 11.3	907.6 ± 13.9	894.8 ± 15.7	900.4 ± 7.9
	EIG70	1502.6 ± 12.6	1514.6 ± 7.9	1507.0 ± 15.6	1524.0 ± 21.7	1509.0 ± 22.0	1514.0 ± 11.4
	EIG80	2591.4 ± 20.0	2616.8 ± 10.8	2597.8 ± 21.3	2622.6 ± 32.3	2609.4 ± 30.6	2611.8 ± 17.2
	EIG90	5130.4 ± 34.7	5182.2 ± 17.4	5146.2 ± 30.1	5180.8 ± 52.6	5175.2 ± 46.3	5171.6 ± 28.7
	EIG95	8632.6 ± 51.8	8707.8 ± 25.8	8652.2 ± 38.2	8700.0 ± 76.3	8705.4 ± 63.7	8688.6 ± 40.6
	EIG98	14,948.4 ± 76.7	15,047.0 ± 36.0	14,968.6 ± 47.1	15,034.8 ± 109.6	15,057.2 ± 89.4	15,016.4 ± 55.8
	EIG99	21,102.8 ± 95.6	21,216.8 ± 43.1	21,125.6 ± 54.2	21,204.6 ± 133.6	21,236.6 ± 109.4	21,176.8 ± 67.0
	All	30,000	30,000	30,000	30,000	30,000	30,000

Q200: number of QTN equal to 200 (less polygenic); Q2000: number of QTN equal to 2000 (more polygenic); H30, H90, H99: heritability scenarios of 0.3, 0.9, and 0.99; EIG*x*: number of largest eigenvalues explaining *x* percent of variance in **G**, which is equivalent to the number of genotyped animals in each set

### Models and analyses

#### Genome-wide associations

Discovery sets were used for GWA (Table 2). Efficient mixed-model association eXpedited (EMMAX) analysis was performed using the Gemma software [18] and the following univariate linear mixed model:

$${\mathbf{y}} = {\mathbf{1}}{\upmu } + {\mathbf{x}}_{{\varvec{i}}} {\mathbf{b}}_{{\varvec{i}}} + {\mathbf{Zu}} + {\mathbf{e}},$$

where ${\mathbf{y}}$ is the vector of phenotypes, ${\upmu }$ is the overall mean, ${\mathbf{x}}_{{\varvec{i}}}$. is the vector of genotypes for the $i{\text{th}}$ SNP, ${\mathbf{b}}_{{\varvec{i}}}$ is the substitution effect of the $i{\text{th}}$ SNP, ${\mathbf{Z}}$ is an incidence matrix for vector ${\mathbf{u}}$, which is the vector of random additive genetic effects, with ${\mathbf{u}}{ }\sim {\text{ N}}\left( {0,{ }{\mathbf{G}}\sigma_{u}^{2} } \right)$, where $\sigma_{u}^{2}$ is the additive genetic variance, and ${\mathbf{e}}$ is the vector of residuals, with ${\mathbf{e}}{ }\sim {\text{ N}}\left( {0,{ }{\mathbf{I}}\sigma_{e}^{2} } \right)$, where $\sigma_{e}^{2}$ is the residual variance, and ${\mathbf{I}}$ is an identity matrix. The ${\mathbf{G}}$ matrix in this step was computed as in Zhou and Stephens [18]:

$${\mathbf{G}} = { }\frac{1}{{n_{s} }}{ }\sum\limits_{{{\text{i}} = 1}}^{{n_{s} }} {\left( {{\mathbf{x}}_{{\mathbf{i}}} - { }{\mathbf{1}}_{{\varvec{n}}} {\overline{\text{x}}}_{{\text{i}}} } \right)\left( {{\mathbf{x}}_{{\mathbf{i}}} - { }{\mathbf{1}}_{{\varvec{n}}} {\overline{\text{x}}}_{{\text{i}}} } \right)^{{\text{T}}} } ,$$

where ${\mathbf{x}}_{{\mathbf{i}}}$ is the $i{\text{th}}$ SNP locus column, $\mathbf{1}_{{\varvec{n}}}$ is the $n \times 1$ vector of 1s, ${\overline{\text{x}}}_{{\text{i}}}$ is the marker sample mean of the $i{\text{th}}$ locus, $n$ and $n_{s}$ are the numbers of genotyped animals and SNPs, respectively. A significance threshold for GWAS was determined by the significance level of 0.05 accounting for multiple testing through Bonferroni correction, which was calculated by 0.05 divided by the number of SNPs and QTN used for the GWA.

### Relationship between the amount of variance explained, sample size, and heritability

The total proportion of genetic variance explained by the identified QTN from GWA was calculated as the sum of the genetic variance explained by each QTN divided by the total additive genetic variance. As QTN effects were given by the simulation, the percentage of genetic variance explained by an individual QTN ($\% Var$) was calculated as:

$$\% Var = 2pq\beta^{2} /\sigma_{u}^{2} ,$$

where $p$ and $q$ are the major and minor allele frequencies of the QTN, $\beta$ is the QTN effect, and $\sigma_{u}^{2}$ is the total additive genetic variance of the model. Therefore, the additive genetic variance differed according to the heritability applied in each scenario.

In the current study, we estimated the corresponding sample size based on the total proportion of variance explained by the identified QTN in each heritability scenario. To be more precise, each corresponding sample size is estimated to achieve a particular proportion of variance explained in each heritability scenario. This estimation was done by local polynomial regression [19] using the ‘loess’ and ‘approx’ functions in R by regressing total proportion of variance explained by the identified QTN on the sample size, and the resulting sample size was represented by ${\text{SS}}_{{{\text{pol}}}}$. We estimated the sample size using H30 as a benchmark since it is the lowest heritability scenario. This helped us identify how many samples are needed for the GWA under different heritability scenarios.

### Preselection of variants for genomic prediction

Different numbers of variants were selected from the GWA and were included in the 50k SNP panel for genomic prediction. Each ‘QTN’ scenario had a specific number of selected variants based on the order of the p-values (TOP*v*), without considering statistical significance. For Q200, *v* was equal to 10, 50, 100, 200, or 400, whereas, for Q2000, it was equal to 10, 100, 500, 1000, 2000, or 4000. We considered one additional scenario by selecting only the significant variants based on the statistical significance using Bonferroni corrected p-values (SIG).

### Genomic prediction

Training and test sets were used only for genomic prediction. A linear mixed model was used to compute genomic prediction:

$${\mathbf{y}} = {\mathbf{1}}{\upmu } + {\mathbf{Zu}} + {\mathbf{e}},$$

where ${\mathbf{y}}$ is the vector of phenotypes, ${\upmu }$ is the overall mean, ${\mathbf{Z}}$ is the incidence matrix for ${\mathbf{u}}$, which is the vector of random additive genetic effects, with ${\mathbf{u}}{ }\sim {\text{ N}}\left( {0,{ }{\mathbf{H}}\sigma_{u}^{2} } \right)$, where $\sigma_{u}^{2}$ is the additive genetic variance and ${\mathbf{H}}$ is the realized relationship matrix; ${\mathbf{e}}$ is the vector of residuals, with ${\mathbf{e}}{ }\sim {\text{ N}}\left( {0,{ }{\mathbf{I}}\sigma_{e}^{2} } \right)$, where $\sigma_{e}^{2}$ is the residual variance. These variances were from the simulations, so they were scenario-specific. Genomic prediction was performed using ssGBLUP in the BLUPF90 family of programs [17]. For the mixed model equations in ssGBLUP, ${\mathbf{H}}^{ - 1}$ combines pedigree and genomic relationships [20]:

$${\mathbf{H}}^{ - 1} = {\mathbf{A}}^{ - 1} + \left[ {\begin{array}{*{20}c} 0 & 0 \\ 0 & {{\mathbf{G}}^{ - 1} - { }{\mathbf{A}}_{22}^{ - 1} } \\ \end{array} } \right],$$

where ${\mathbf{G}}^{ - 1}$ is the inverse of the genomic relationship matrix and ${\mathbf{A}}_{22}^{ - 1}$ is the inverse of the pedigree relationship matrix for the genotyped animals. The ${\mathbf{G}}$ matrix was built as in the first method of VanRaden [21]:

$${\mathbf{G}} = { }\frac{{{\mathbf{MM^{\prime}}}}}{{2\sum {\text{p}}_{{\text{i}}} \left( {1 - {\text{p}}_{{\text{i}}} } \right)}},$$

where ${\mathbf{M}}$ is the matrix of genotypes centered for the current allele frequencies, ${\text{p}}_{{\text{i}}}$ is the minor allele frequency of the $i{\text{th}}$ SNP. To avoid singularity issues, ${\mathbf{G}}$ was blended with 5% of ${\mathbf{A}}_{22}$. In this study, the dimension of ${\mathbf{G}}$ and ${\mathbf{A}}_{22}$ differed based on training set scenarios (EIG*x*, Table 2). We compared results from ssGBLUP with those from pedigree-based BLUP (PBLUP).

### Validation of genomic predictions

In each scenario, prediction accuracy was calculated as the correlation between TBV and GEBV. The regression coefficient (b_1_) of TBV on GEBV was used as an indicator of inflation (i.e., b_1_ < 1) or deflation (i.e., b_1_ > 1) of GEBV.

## Results

### Identification of variants

**Fig. 1:**
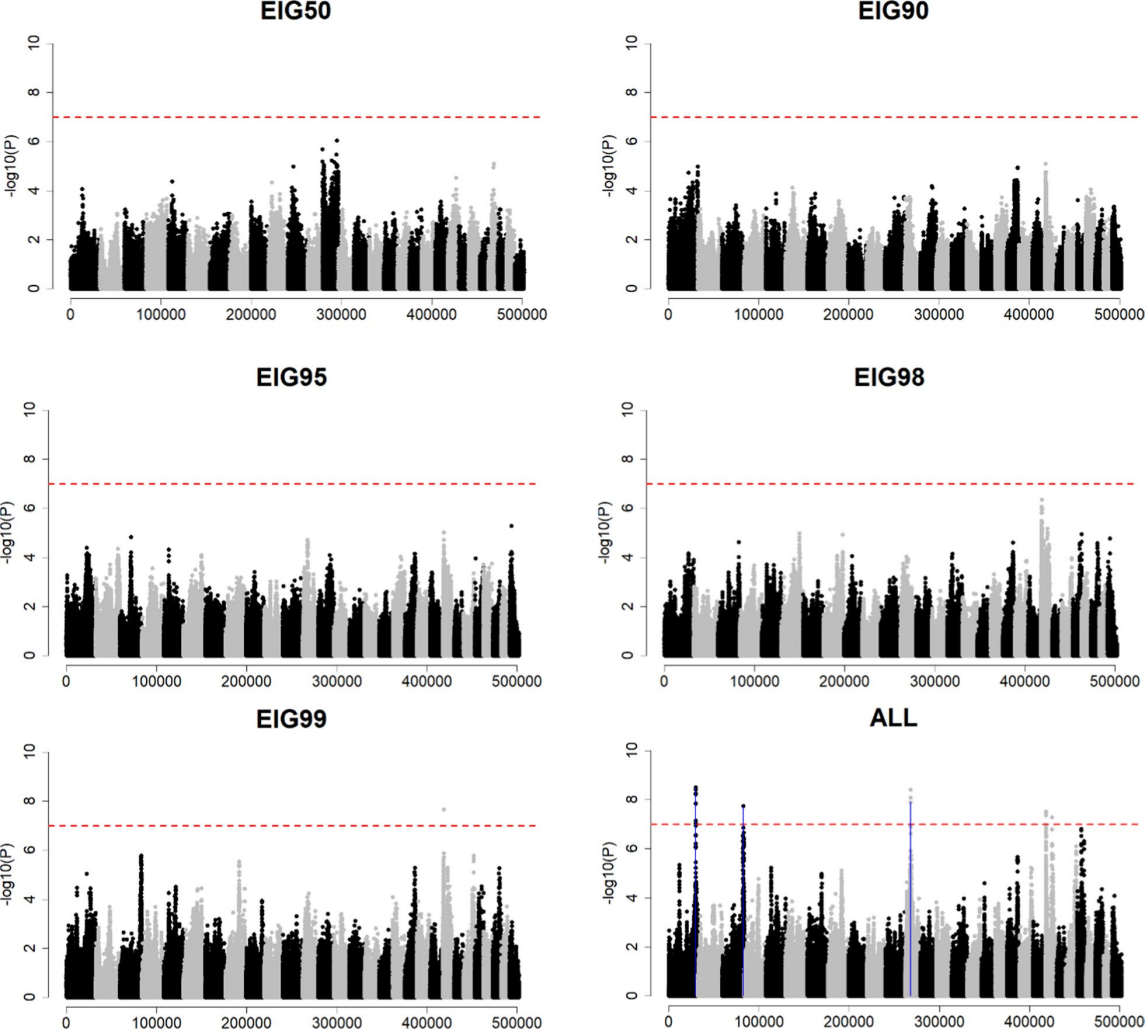
Genome-wide association using sample size based on the percentage of variance explained by eigenvalues (EIG*x*, with *x* = 50%, 90%, 95%, 98%, and 99%) or all genotyped individuals (ALL) when Ne was 20 (*Ne*20), QTN was 2000 (Q2000) and h^2^ of 0.3 (H30). The x-axis and y-axis indicate the number of variants and − log10(p-value), respectively. The red horizontal dashed line represents the Bonferroni correction threshold. Blue vertical lines point out the QTN position, which was identified as significant

**Fig. 2:**
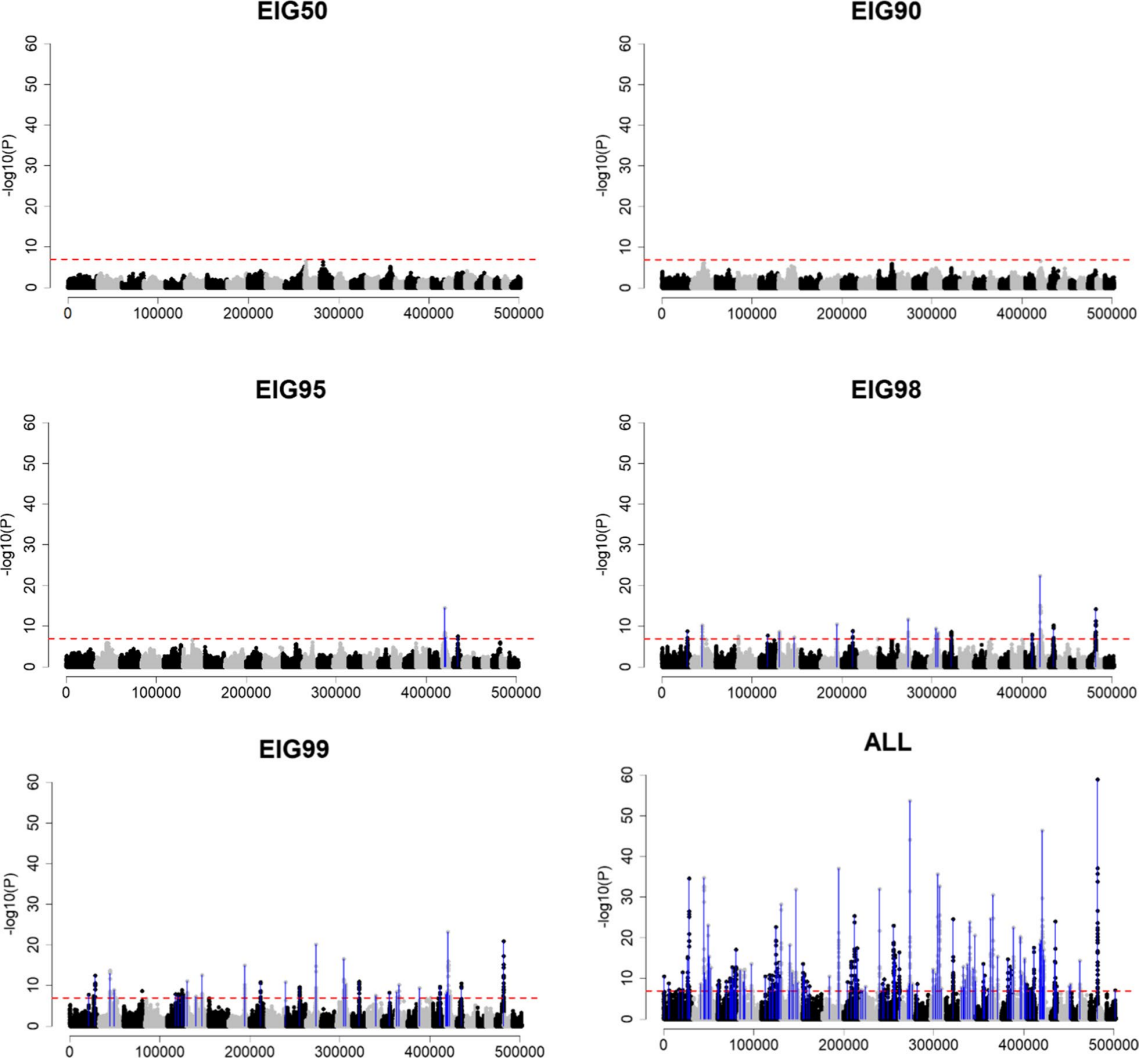
Genome-wide association using sample size based on the percentage of variance explained by eigenvalues (EIG*x*, with *x* = 50%, 90%, 95%, 98%, and 99%) or all genotyped individuals (ALL) when Ne was 20 (*Ne*20), QTN was 2000 (Q2000) and h^2^ of 0.99 (H99). The x-axis and y-axis indicate the number of variants and − log10(p-value), respectively. The red horizontal dashed line represents the Bonferroni correction threshold. Blue vertical lines point out the QTN position, which was identified as significant

**Fig. 3:**
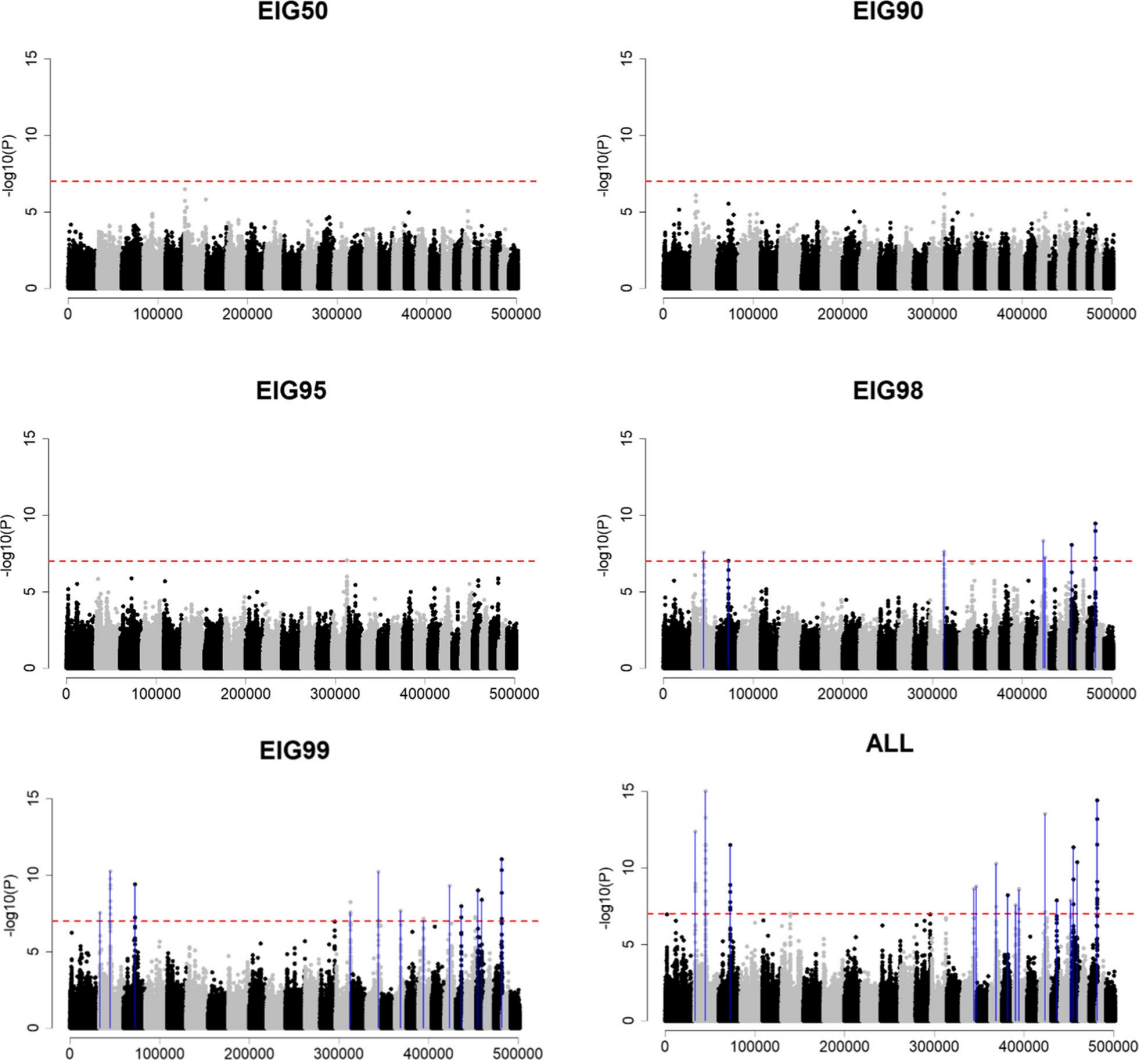
Genome-wide association using sample size based on the percentage of variance explained by eigenvalues (EIG*x*, with *x* = 50%, 90%, 95%, 98%, and 99%) or all genotyped individuals (ALL) when Ne was 200 (*Ne*200), QTN was 2000 (Q2000) and h^2^ of 0.3 (H30). The x-axis and y-axis indicate the number of variants and − log10(p-value), respectively. The red horizontal dashed line represents the Bonferroni correction threshold. Blue vertical lines point out the QTN position, which was identified as significant

**Fig. 4:**
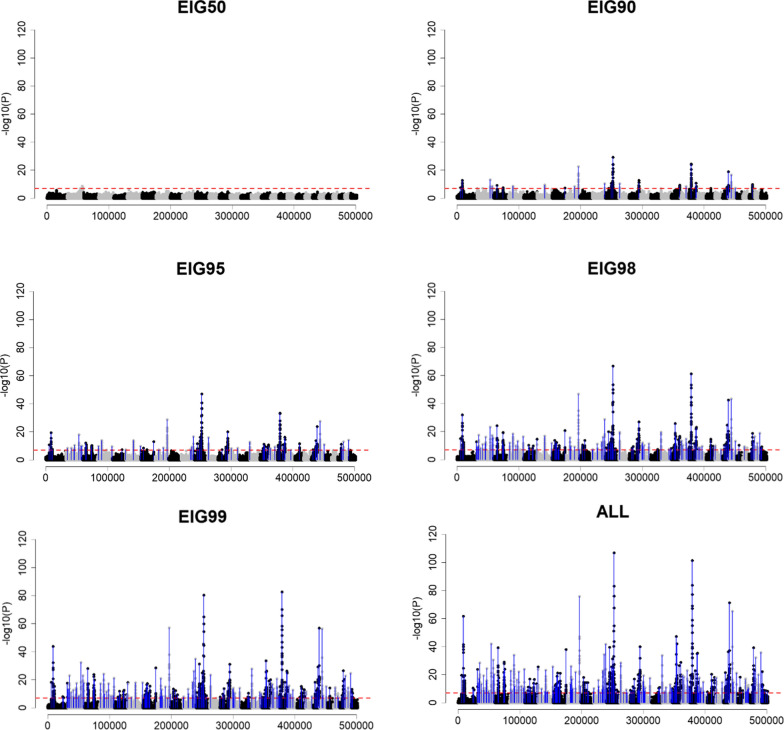
Genome-wide association using sample size based on the percentage of variance explained by eigenvalues (EIG*x*, with *x* = 50%, 90%, 95%, 98%, and 99%) or all genotyped individuals (ALL) when Ne was 200 (*Ne*200), QTN was 2000 (Q2000) and h^2^ of 0.99 (H99). The x-axis and y-axis indicate the number of variants and − log10(p-value), respectively. The red horizontal dashed line represents the Bonferroni correction threshold. Blue vertical lines point out the QTN position, which was identified as significant

The results of the GWA analyses are shown in Figs. 1, 2, 3, and 4. Because each simulated replication generated different QTN positions and effects, the results of only one replicate are presented. Since most quantitative traits are highly polygenic, only the results of the scenarios Q2000 with H30 and H99 and the two *Ne* are shown. In addition, the GWA results with EIG60, EIG70, and EIG80 are not included in Figs. 1, 2, 3, and 4 because very few significant peaks were observed for these EIG*x* scenarios lower than EIG90. These results and those obtained with H90, Q200, and EIG60, EIG70, and EIG80 are provided in Additional file 13: Fig. S1a–l. The numbers of significantly identified QTN, SNPs, and the variance explained by QTN across the five replicates are presented in Additional file 14: Table S1a–d. In the scenario of *Ne*20 Q2000 H30, using EIG50, EIG90, EIG95, EIG98, and EIG99, the sample size for GWA was not sufficient to significantly detect any QTN (Fig. 1). However, when the sample size increased to 30,000 (i.e., ALL), three significant QTN were detected. In contrast, the high heritability scenario (H99) increased the ability to identify simulated QTN correctly (Fig. 2). With EIG95, three QTN were identified, and as sample size increased to EIG98, EIG99, and ALL, 17, 33, and 142 QTN were identified, respectively.

Compared to an *Ne* of 20, the results were different with an *Ne* of 200, when contrasting EIG*x* were used as the sample size (Figs. 3 and 4). Although EIG50, EIG90, and EIG95 were not sufficient to identify any QTN in *Ne*200 Q2000 H30 when the number of genotyped animals increased to EIG98, seven QTN were identified (Fig. 3) and when it increased to EIG99 and ALL even more QTN were identified, and the GWA resolution was improved. In the scenario of *Ne*200 Q2000 H99, EIG90 is an adequate sample size to detect the QTN with the largest effect size (Fig. 4). For this scenario, EIG98 provided a clear resolution, similar to EIG99 and ALL. It is important to note that the number of largest eigenvalues explaining a certain proportion of the variance in ${\mathbf{G}}$ was different for *Ne*20 and *Ne*200 (Table 2). When all available genotyped animals were used (i.e., ALL), a larger number of significant QTN were identified in GWA with *Ne*200 than with *Ne*20. For example, in *Ne*20 Q2000 H30, the three identified QTN captured 3.5% of the additive genetic variance (see Additional file 14: Table S1b), whereas, in *Ne*200 Q2000 H30, 18 identified QTN captured 17.0% (see Additional file 14: Table S1d). With both *Ne* values, H99 was more efficient to detect QTN than H30. Fewer genotyped animals were required to identify the simulated QTN for a less polygenic trait (Q200) than for a more polygenic trait (Q2000).

### Relationship between the amount of variance explained, sample size, and heritability

**Fig. 5:**
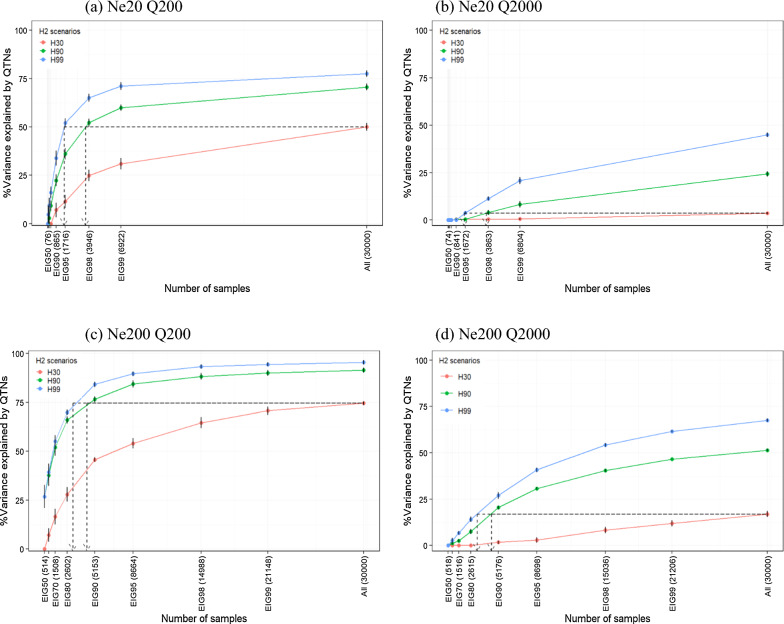
Total variance explained by significant QTN across the different sample sizes and heritabilities. Each x-axis and y-axis indicate the number of genotyped animals (EIG*x* scenarios) and percentage of total variance explained by identified QTN for three heritability scenarios (0.3 in H30, 0.9 in H90, and 0.99 in H99)

The average proportion of variance explained by the identified QTN and the respective standard errors (SE) from five replicates are shown in Fig. 5. All the results for the number of identified significant QTN and SNPs and the proportion of variance explained are in Additional file 14: Table S1a–d. Since many scenarios were considered, we will describe the results in the following order: (1) sample size (EIGx), (2) heritability, and (3) *Ne*. First, as the sample size increased, the proportion of variance explained by the identified QTN increased regardless of the heritability, *Ne*, and the number of QTN. Second, when the proportion of variance explained was analyzed according to heritability, high heritability scenarios were more efficient to identify QTN. For example, when all genotyped animals (i.e., ALL) were used in the scenario *Ne*20 Q200 H30, it was possible to identify QTN explaining 50.1% of the variance (Fig. 5a). However, H90 and H99 identified QTN explaining 70.7 and 77.6% of the variance, respectively, with the same number of genotyped animals. For *Ne*20 Q2000 H30 and H99 (Fig. 5b), identified QTN explained 3.5 and 45.0% of the variance, respectively. A similar pattern was observed in *Ne*200 (Fig. 5c, d), which also showed that higher heritability scenarios were more efficient to identify QTN. Third, the comparison of the results with *Ne*20 and *Ne*200 showed that the identified QTN explained a greater proportion of variance in the *Ne*200 scenarios (Fig. 5c, d) than in the *Ne*20 scenarios (Fig. 5a, b). For example, the maximum proportion of variance explained by QTN with *Ne*20 Q200 H99 and *Ne*20 Q2000 H99 was 77.6 and 45.0% (Fig. 5a, b), whereas the *Ne*200 Q200 H99 and *Ne*200 Q2000 H99 showed 95.5 and 67.6% (Fig. 5c, d). Furthermore, one remarkable result was that in the scenario with a less polygenic trait, with EIG98 and EIG99, the proportion of variance explained by the identified QTN was similar to that with ALL (Fig. 5a, c) compared to the scenario with a more polygenic trait (Fig. 5b, c). In the *Ne*20 Q200 H99 scenario, the EIG98, EIG99, and ALL identified QTN explaining 65.2, 71.2, and 77.6% of the variance, respectively (Fig. 5a). In the *Ne*20 Q2000 H99 scenario, the EIG98, EIG99, and ALL identified QTN explaining 11.3, 20.8, and 45.0% of the variance (Fig. 5b), respectively; therefore, the proportion of variance explained increased by almost fourfold from EIG98 to ALL, whereas this increase was only 20% with Q200. In the *Ne*200 Q200 H99 scenario, EIG98, EIG99, and ALL detected QTN explaining 93.2, 94.4, and 95.5% of the variance, respectively (Fig. 5c). Even for the more polygenic scenario (*Ne*200 Q2000 H99), EIG98, EIG99, and ALL captured QTN explaining 54.3, 61.6, and 67.6%, respectively (Fig. 5d). We observed similar patterns for the other two heritability scenarios (H30 and H90) (see Fig. 5c, d).

To investigate the corresponding sample size in the different heritability scenarios that would achieve a particular level of variance explained, we used the H30 as a benchmark. The dashed arrows in Fig. 5 show the estimated sample size for H90 and H99 when the largest discovery set (ALL, N = 30,000) was used for H30 through local polynomial regression. Table 3 presents the percentage of variance explained when the largest discovery set (ALL, N = 30,000) was used for H30 and the estimated sample sizes required for H90 and H99 to achieve an equivalent proportion of variance explained as that attained by H30 using ALL. All other estimated sample sizes based on the different EIG*x* as a benchmark are in Additional file 14: Table S2a–d. Table 3 shows that for scenarios *Ne*20 Q200 H90 and *Ne*20 Q200 H99, 3626 and 1622 genotyped animals (SS_pol) are required to achieve the same level of genetic variance explained (50.1%) by scenario *Ne*20 Q200 H30 with ALL. The scenarios *Ne*20 Q2000 H90 and *Ne*20 Q2000 H99 required 3652 and 1599 genotyped animals (SS_pol) to identify QTN that explained 3.5% of the variance, which is equivalent to the level achieved in scenario *Ne*20 Q2000 H30 with ALL. We observed a similar pattern when *Ne* was 200. A remarkable difference between results from *Ne*20 and *Ne*200 was that the sample sizes required to achieve an equivalent level of variance explained by the QTN using ALL in H30 were comparable to the range of EIG90 to EIG98 in *Ne*20, but EIG80 to EIG90 in *Ne*200 when considering H90 and H99 (${\text{EIGx}}_{{{\text{SSpol}}}}$).

**Table 3:** Estimated sample size based on local polynomial regression using ‘ALL’ and ‘H30’ as benchmark

Scenario	Heritability	%Var	${\text{SS}}_{{{\text{pol}}}}$	${\text{EIGx}}_{{{\text{SSpol}}}}$
*Ne*20 Q200	H30 (ALL)	50.1		
	H90	50.1	3626	EIG95–98 (1719–3952)
	H99	50.1	1622	EIG90–95 (871–1728)
*Ne*20 Q2000	H30 (ALL)	3.5		
	H90	3.5	3652	EIG95–98 (1674–3854)
	H99	3.5	1599	EIG90–95 (833–1658)
*Ne*200 Q200	H30 (ALL)	74.5		
	H90	74.5	4458	EIG80–90 (2617–5182)
	H99	74.5	3153	EIG80–90 (2598–5146)
*Ne*200 Q2000	H30 (ALL)	17.0		
	H90	17.0	4526	EIG80–90 (2609–5175)
	H99	17.0	3206	EIG80–90 (2612–5172)

Dashed arrows in Fig. 5 correspond to the estimated sample size for each line of the H90 and H99 scenarios, which were obtained using local polynomial regression. These values are referred to as SS_pol

%Var: percentage of variance explained by significantly identified QTN; ${\text{SS}}_{{{\text{pol}}}}$: estimated sample size using local polynomial regression; ${\text{EIGx}}_{{{\text{SSpol}}}}$: EIGx scenario range including ${\text{SS}}_{{{\text{pol}}}}$

### Genomic predictions

Initially, we assessed the potential bias of genomic prediction by using the same set of genotyped animals for both the discovery and training sets. Notably, using different groups of genotyped animals for the discovery and training sets produced less inflation of GEBV than using the same animals for both processes (results not shown). Therefore, the genomic prediction analyses were performed with training animals that were different from those in the discovery set.

**Fig. 6:**
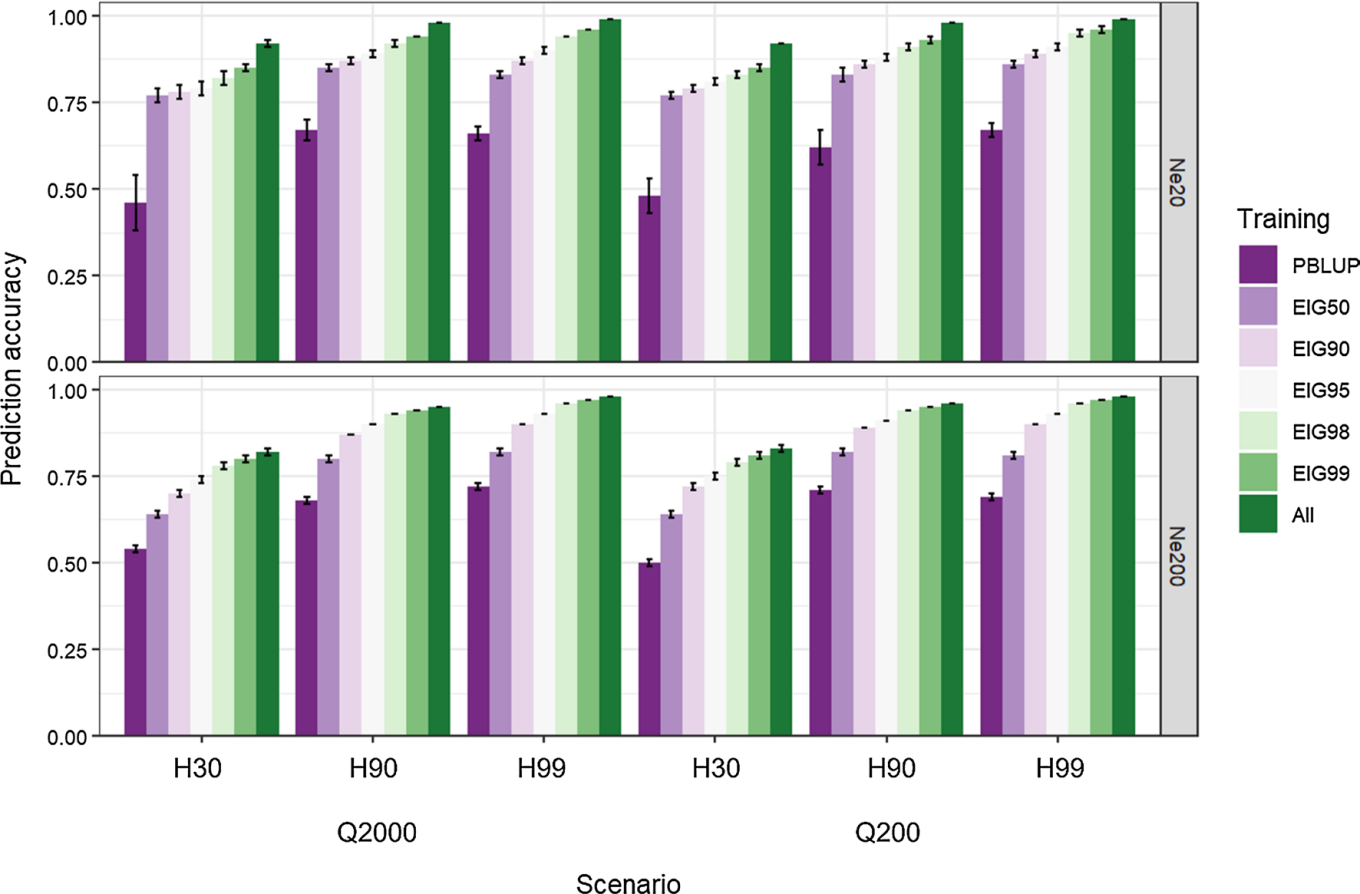
Prediction accuracy (correlation between true and estimated breeding values) for PBLUP or ssGBLUP using training size based on the percentage of variance explained by eigenvalues (EIG*x*, with *x* = 50%, 90%, 95%, 98%, and 99%) or all genotyped individuals (ALL) for different *Ne*, QTN, and heritability. Results in each training set are the average of all genotyped scenarios: 50k, TOP10, TOP50, TOP100, TOP200, TOP400, and ‘SIG’ for Q200 and 50k, TOP10, TOP100, TOP500, TOP1000, TOP2000, TOP4000, and ‘SIG’ for Q2000 scenarios except for PBLUP

**Fig. 7:**
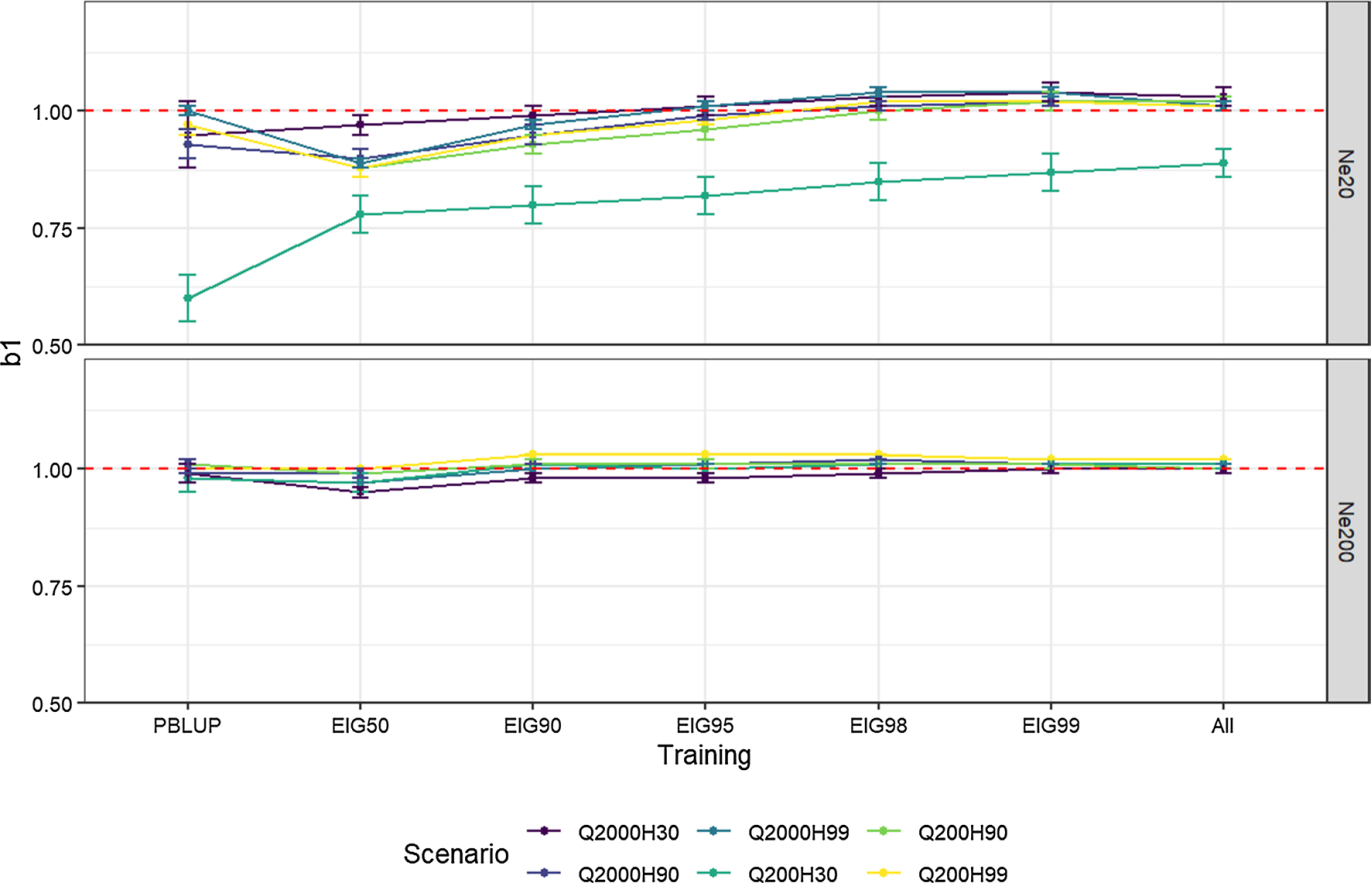
Regression coefficients (b1) of true on estimated breeding values from PBLUP or ssGBLUP using training size based on the percentage of variance explained by eigenvalues (EIGx, with x = 50%, 90%, 95%, 98%, and 99%) or all genotyped individuals (ALL) for different *Ne*, QTN, and heritability. Results in each training set are the average of all genotyped scenarios: 50k, TOP10, TOP50, TOP100, TOP200, TOP400, and ‘SIG’ for Q200 and 50k, TOP10, TOP100, TOP500, TOP1000, TOP2000, TOP4000, and ‘SIG’ for Q2000 scenarios except for PBLUP

Figures 6 and 7 present the average prediction accuracy and inflation/deflation indicator of GEBV (b_1_), respectively, with their corresponding standard errors. Those accuracies and b_1_ were calculated as the average of all genotyped scenarios: 50k, TOP10, TOP50, TOP100, TOP200, TOP400, and ‘SIG’ for the Q200 and 50k, TOP10, TOP100, TOP500, TOP1000, TOP2000, TOP4000, and ‘SIG’ for the Q2000 scenarios. Similar to Figs. 1, 2, 3, and 4, EIG60, EIG70, and EIG80 were excluded from Figs. 6 and 7 due to the insignificance of their results (results now shown). Figure 6 shows the prediction accuracy according to the number of QTN (Q2000 or Q200), trait heritability (H30, H90, and H99), and training data scenarios (EIGx and ALL). Results for all other scenarios are in Additional file 15: Table S3. In this study, we also examined the prediction accuracies based on EBV computed without genomic information, using a method referred to as PBLUP. We investigated the effect of the size of the training set on prediction accuracy and observed different patterns between populations with *Ne* of 20 and 200. Our results for *Ne*200 showed that as the size of the training set increased from EIG50 to EIG90, prediction accuracy also increased, as demonstrated in Fig. 6. For instance, when the training set was upgraded from EIG50 to EIG90 in the scenario *Ne*200 Q200 H30, the prediction accuracy increased by 0.08, from 0.64 to 0.72. A similar pattern was observed in *Ne*200 Q2000 H30, where a gain of 0.07 (0.64 to 0.71) was achieved when the training set was upgraded from EIG50 to EIG90. However, in the same scenarios with *Ne*20, the gain was only about 0.02, at most. Generally, *Ne*20 showed greater prediction accuracy than *Ne*200. For example, when the smallest sample size (EIG50) was used for *Ne*20 Q2000 H30, H90, and H99 scenarios, prediction accuracies were 0.77, 0.85, and 0.83, respectively. Conversely, the same scenarios with *Ne*200 showed lower prediction accuracies of 0.64, 0.79, and 0.82, respectively. However, this difference became smaller with the largest sample size (ALL). Similar patterns were observed for the Q200 scenarios. Prediction accuracies were highly influenced by the heritability of the trait, particularly in populations with a larger effective size (*Ne*200). For instance, when EIG50 was used for *Ne*200 Q200 H30, H90, and H99 scenarios, the prediction accuracies were 0.64, 0.82, and 0.81, respectively. Even with the largest training set (ALL), prediction accuracies were 0.85, 0.97, and 0.99, following the same order. Similar trends were also observed for *Ne*200 Q2000 scenarios. PBLUP always showed lower prediction accuracies than the other scenarios with ssGBLUP.

The regression coefficients (b_1_) are presented in Fig. 7, along with their SE. When the size of the training set was small, less inflation was observed when *Ne* was 200 than 20. However, using a large training set reduced the inflation for both effective population sizes. When ALL was used for training, all scenarios had b_1_ values close to 1, except for the *Ne*20 Q200 H30 scenario. In addition, this scenario exhibited the largest SE between the replicates (~ 0.04) compared to other scenarios. We also found greater inflation of EBV and more variation between the models based on PBLUP with *Ne*20 compared to *Ne*200.

**Fig. 8:**
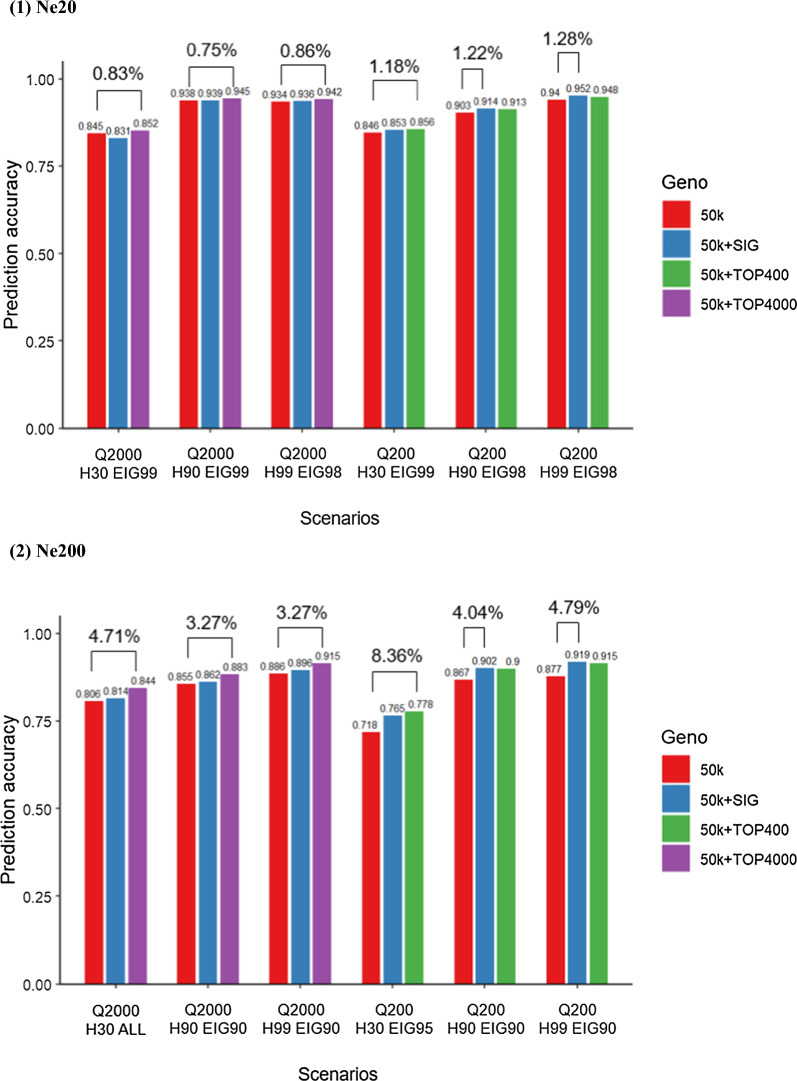
Prediction accuracy using 50k SNP with and without adding SNPs selected from GWA (TOP*v* with *v* being the number of SNPs with the smallest p-values) and significant SNPs from GWA (SIG). Only the scenarios with the maximum gain (%) in prediction accuracy are shown

Figure 8 displays the prediction accuracy of using 50k compared to 50k plus SIG, TOP400 (Q200), and TOP4000 (Q2000), along with the percentage of gain achieved by adding selected variants. Among all the analyses that combined the 50k and the top SNPs, in Fig. 8 we present only the scenarios that exhibited the largest differences among the EIG scenarios, as the changes were not significant across all analyses. Overall, the percentage of gain was generally higher with *Ne*200 (ranging from 3.27 to 8.36%) than with *Ne*20 (ranging from 0.75 to 1.28%). Moreover, Q200 showed a higher percentage of gain than Q2000 in both *Ne* scenarios. Notably, the maximum accuracy gain was typically observed when the largest number of top SNPs (TOP400 for Q200 and TOP4000 for Q2000) was added to 50k chip data, which represented twice the number of simulated QTN than by adding SIG. However, in the scenarios *Ne*20 Q200 H90, *Ne*20 Q200 H99, *Ne*200 Q200 H90, and *Ne*200 Q200 H99, the highest increase in accuracy was observed when using 50k plus SIG. This is probably because identifying significant QTN was easier for a less polygenic trait (Q200) with H90 and H99 but more challenging in Q2000 or for a low heritability trait (H30).

## Discussion

In this study, we conducted a thorough investigation of the impact of various sample sizes in GWA, which followed the concept of dimensionality of genomic information, the implications of using different trait heritabilities (to mimic GEBV reliability) in GWA, and the inclusion of preselected variants into a typical 50k SNP panel using ssGBLUP. Our analysis provided valuable insights into how different data structures can affect the performance of GWA and genomic prediction under the ssGBLUP framework. We observed that the concept of limited dimensionality of genomic information [4] could be a helpful indicator of the number of genotyped animals required for GWA, depending on *Ne*, *Me*, the number of QTN, and the trait heritability (i.e., reliability of GEBV). According to our findings, a sample size with the same number of genotyped animals as that of EIG98 was suitable for detecting genetic variants in GWA, particularly in populations characterized by large *Ne* (Fig. 5c, d). This finding holds true, especially when the genotyped animals had a high GEBV reliability. In addition, incorporating selected variants identified through GWA into the 50k SNP chip can enhance prediction accuracy when an appropriate training set size is used (i.e., EIG98 in *Ne*20 and EIG90 in *Ne*200).

### GWA—preselection of variants for genomic prediction

The most common approach for implementing genomic prediction using sequence data involves a three-step workflow. First, significant variants are preselected. Next, these selected variants are incorporated into commercial SNP chip data (i.e., 50k), or separate genomic matrices are fitted in the model [9, 22, 23]. Finally, the genomic prediction performance is compared to that obtained using a benchmark SNP chip. While several studies have attempted to improve genomic prediction using sequence data, both with simulated and real datasets, the literature lacks consistent conclusions regarding the advantages of using sequence data [9, 22, 24, 25]. This may be influenced by various factors, including but not limited to the species under study, the genetic architecture of the trait being examined, the size of the dataset, and the statistical methods employed. Among those factors, the size of the dataset for discovery, training, and testing sets is the most crucial. Specifically, the size of the variant discovery set is particularly important, as it is the initial step and can significantly affect the outcomes of the entire study. Our current findings suggest that a small number of genotyped animals may not be sufficient to identify the significant SNPs or QTN. This is consistent with the results of Lourenco et al. [26], who used different numbers of genotyped animals (N = 2000 and 25,000) for GWA and observed that the best resolution was achieved when more genotyped animals were used. Similarly, de Las Heras-Saldana et al. [27] highlighted the importance of using a larger dataset for GWA, as it allowed for a better identification of quantitative trait loci (QTL) regions for carcass traits in Hanwoo cattle.

With the current increase in the number of genotyped animals in many species, such as the 6.4 million U.S. Holsteins (https://queries.uscdcb.com/Genotype/cur_freq.html), and approximately 1.3 million American Angus cattle (A. Garcia, American Angus Association, Saint Joseph, MO, personal communication) as of April 2023, it is crucial to determine the minimum number of genotyped animals required to detect significant variants. Our findings indicate that a sample size with at least the same number of genotyped animals as that of EIG98 could effectively identify the most informative QTN when *Ne* is large. In populations with *Ne*200, using EIG99 or all available genotyped animals only slightly improved the detection of significant QTN beyond using EIG98 for both Q200 and Q2000 scenarios. For example, we observed that the average increase in the proportion of variance explained by identified significant QTN when using ALL (all available genotyped animals) as opposed to EIG98 was 8.1% in populations with *Ne*200 and 18.9% in populations with *Ne*20. However, it is important to note that some scenarios had very small variances explained at EIG98, such as 0.3% for *Ne*20 Q2000 H30 and 8.3% for *Ne*200 Q2000 H30. While the percentage increase in variance explained may appear large in some scenarios with small variances explained at EIG98, the limited impact on predictions may not justify genotyping many more animals. (i.e., EIG98 vs. ALL). This result could be helpful for both small and large genotyped populations with large *Ne*. In breeding populations with limited resources, the number of animals that can be genotyped or sequenced may be restricted. In such scenarios, determining the required sample size can aid in cost-effective genotyping or sequencing practices. Our study suggests that in populations with large *Ne* (*Ne*200), it is not necessary to use all available animals for variant discovery. Instead, a balanced dataset should be constructed for discovery, training, and testing to avoid biases and optimize the ability to detect significant variants. In populations with small *Ne* (*Ne*20) and highly polygenic traits (Q2000), most sample sizes were not sufficient to identify any significant QTN until it reached ALL and EIG98 for H30 and H90, respectively. However, the availability of more information on genotyped animals, as seen in H99, allowed the identification of a few QTN with a sample size equivalent to EIG95, indicating the importance of sample size and amount of information for identifying significant signals in livestock species, such as chicken and pigs, which have smaller *Ne* (32–48) compared to cattle [28]. Therefore, a sample size smaller than ALL would not be sufficient to detect significant signals in species with small *Ne*. Gozalo-Marcilla et al. [29] performed a large-scale GWA for backfat thickness in pigs using 15k to 55k genotyped animals. They identified 264 significant SNPs across eight different lines for traits with moderate to high heritability (0.30–0.58). As backfat thickness is known to have a polygenic architecture (more than 1400 QTL associated backfat thickness are reported in https://www.animalgenome.org/QTLdb), their discovery is supported by our findings in populations with small *Ne* (i.e., 20), moderate to high heritability (0.3 to 0.9), and more polygenic traits (number of QTN = 2000) when using 30k genotyped animals for GWA.

### GWA—limited dimensionality of genomic information and trait heritability

Pocrnic et al. [4] described the number of largest eigenvalues explaining a certain proportion of variance of ${\mathbf{G}}$ as a function of *Ne* and genome length in Morgans, such that EIG90 $\approx$ *NeL*, EIG95 $\approx$ 2*NeL*, and EIG98 $\approx$ 4*NeL*. Stam [3] expressed the expected number of independent chromosome segments as *Me* = 4*NeL*. Given that *Ne* and *Me* are proportional, a smaller *Ne* indicates a reduced *Me*, which reflects a strong LD between variants due to the close genetic relatedness between individuals. The impact of *Ne* on the performance of GWA has also been reported [26, 30]. Our study demonstrated that, when the same number of genotyped animals was used (ALL), *Ne*200 outperformed *Ne*20 in identifying significant QTN that explained a higher proportion of the genetic variance. This finding is generalizable across all heritability and QTN scenarios investigated. The reason for this could be attributed to shorter chromosome segments and weaker LD between the QTN and SNPs in the *Ne*200 compared to the *Ne*20 scenario. Pinpointing QTN is more challenging in the *Ne*20 scenario because multiple SNPs may capture the QTN signal. The difficulty of capturing significant QTN in the *Ne*20 scenario is likely due to the strong relationships between the SNPs and QTN, which have been established by a highly structured population across generations. Therefore, identifying the true causative variant in smaller populations is a complex task. In general, the *Ne* of farm animals, including chickens, pigs, dairy, and beef cattle, is less than 200 and can range from 40 to 150 [28]. Consequently, the current findings of our study could provide valuable information for future GWA studies in these species. However, it is important to acknowledge that identifying all significant variants is not guaranteed, as most traits in farm animals are polygenic, and most causal variants exhibit minor effects. For example, even with the largest sample size in our study (ALL, N = 30k), identifying QTN with very small effects was not possible due to limited statistical power. Misztal et al. [12] demonstrated that even when all simulated QTN had identical effects, the GWA in a population with an *Ne* of 600 and a sample size of 6000 could not identify all QTN. In a population with an *Ne* of 60, a sample size that was three times larger resulted in the detection of more true signals, but still fewer than what was detected in a population with an *Ne* of 600. The same authors argued that populations with smaller *Ne* required more data to overcome noise and capture actual signals.

In this study, we investigated the effectiveness of GWA for variant discovery when genotyped animals had more or less information for the computation of GEBV. This was done by varying the trait heritability. Our results indicated that scenarios with a high heritability trait captured more significant QTN explaining a larger portion of additive genetic variance, regardless of the number of QTN, *Ne*, *Me*, and sample size.

In GBLUP or ssGBLUP, SNP effects (${\hat{\mathbf{a}}}$) can be calculated as ${\hat{\mathbf{a}}}|{\hat{\mathbf{u}}} = k{\mathbf{DM^{\prime}G}}^{ - 1} {\hat{\mathbf{u}}}$ [13] and the p-value for SNP $i$ is obtained as $p{\text{-}}value_{i} = 2\left( {1 - {\Phi }\left( {\left| {\frac{{{\hat{\text{a}}}_{i} }}{{sd\left( {{\hat{\text{a}}}_{i} } \right)}}} \right|} \right)} \right)$ [31, 32]. Therefore, it was assumed that high-reliability ${\hat{\mathbf{u}}}$ could yield reliable SNP effects, which results in a smaller standard deviation of SNP effects and generates more accurate p-values. Our findings support this assumption, suggesting that high-reliability GEBV can improve the accuracy of variant discovery and downstream analyses. In a study by Takeda et al. [33], no significant differences were observed in the power to detect QTL when simulated heritability values of 0.2 and 0.5 were compared. However, it was noted that QTL detection improved with an increasing number of phenotyped progenies (N = 1500, 4500, and 9000), which led to a higher reliability of GEBV. In addition, van den Berg et al. [34] reported a decrease in the number of false positive QTL with increasing heritability and more records.

In our study, estimating the required sample sizes for GWA based on regressing total proportion of variance explained by the identified QTN on the sample size was useful to assess the appropriate sample size based on the average reliability of EBV in the set of animals available for GWA. Overall, our findings indicate that smaller sample sizes are adequate for identifying QTN that explain a specific proportion of variance when animals with a high EBV reliability, i.e., in high heritability scenarios, are used. These estimates are in Table 3 and Additional file 14: Table S2a–d, which provide specific numbers and ranges of sample size that can be applied to real data. To determine the necessary sample size for GWA, one could use these estimations by considering the number of animals that can be genotyped or sequenced, the reliability of their EBV, the desired proportion of variance explained by prospective causative variants (i.e., possible QTN), *Ne*, and *Me*. Future research of interest would be on associating those factors and deriving an equation to estimate the suitable sample size for GWA.

### Genomic prediction

In general, genomic prediction accuracy in this study improved as training data size increased, and combining selected variants to a 50k SNP panel enhanced the accuracy when the genomic prediction was performed with the suitable training set sizes. However, the magnitude of this improvement was limited, where only a minor (< 1.0%) increase in accuracy was seen when using training sets with the number of genotyped animals equal to EIG50 to EIG70 for *Ne*20. Several studies have demonstrated that increasing the number of animals in training sets improved the accuracy of genomic prediction [35–37]. In contrast, Moser et al. [38] found no improvement in prediction accuracy when the training size increased from 1239 to 1880 in Australian dairy cattle. Therefore, adding a substantial number of genotyped animals to the training set is necessary to improve prediction accuracy. Our study proposes that the size of the training set can be determined by the number of eigenvalues that explain a particular proportion of the variation in ${\mathbf{G}}$. These patterns of improvement were similar when the effective population sizes were 20 or 200 (*Ne*20 and *Ne*200, respectively); however, the prediction accuracies were generally smaller for the *Ne*200 when the same number of genotyped animals was used. Daetwyler et al. [39] demonstrated that the genomic structure of the population (*Ne* and *Me*) has a significant impact on the prediction accuracy of GBLUP. Their study revealed that a smaller *Ne* achieved better accuracy than a larger *Ne*, regardless of the number of QTL when the same number of individuals were used in the training sets.

The selected variants in our study were above the threshold set by a p-value of 0.05 with the Bonferroni correction for multiple testing in GWA. Although this correction is known to be stringent and potentially leads to an increased number of false negatives, we aimed at testing the predictive power of combining the significant variants with a subset of variants based on sample size (TOP*v*). We demonstrated that incorporating a large number of variants (i.e., twice the number of simulated QTN) in the training set improved prediction accuracy by up to 8%. Previous studies have used selected variants from imputed sequence data to improve genomic predictions in single-breed populations. Veerkamp et al. [11] reported that when selected variants were used for genomic prediction, accuracy decreased, and bias increased. However, VanRaden et al. [24] observed an improvement in accuracy by up to 5% when 16k selected variants were added to 60k chip data. In single-breed populations, an improvement in prediction accuracy using selected variants from sequence data could be limited due to long-range LD; thus, precise identification of variants is much more difficult than in multi-breed or across-breed populations [11].

Fragomeni et al. [7] outlined that including causative QTN in the data while not weighting them differently when constructing ${\mathbf{G}}$ in ssGBLUP increased accuracies by 0.04 when the number of QTN was 100 and 1000, which was similar to our results (0.02–0.06). The authors further observed that incorporating weights derived from SNP effects to ${\mathbf{G}}$ improved accuracies by 0.10 and 0.03 for 100 and 1000 QTN scenarios, respectively. This implies that SNP weighting is more beneficial for less polygenic traits. We conducted preliminary tests to compare the performance of genomic prediction using weighted ssGBLUP (WssGBLUP) and ssGBLUP and found only minor differences between the two methods. The key difference between ssGBLUP and WssGBLUP is that ssGBLUP assumes that all SNPs explain the same amount of genetic variance, whereas WssGBLUP assigns different variances for each SNP [13]. Generally, weighting ${\mathbf{G}}$ may not increase the accuracy of genomic predictions but may improve the GWA resolution, especially in GBLUP-based models for small and large genotyped populations [13, 26].

Our study has shown that when the number of genotyped animals used corresponded at least to the number of eigenvalues explaining 98% of the variation in ${\mathbf{G}}$ for large effective population sizes (i.e., *Ne*200) improved the resolution of variant detection. This suggests that a precise detection of the most significant variants is feasible when the number of genotyped animals for discovery is close to the expected number of independent chromosome segments (*Me*). Using a larger sample size marginally increased the resolution of GWA studies. The genomic information available for such studies is limited in its dimensionality and can be quantified in various ways, such as the number of non-redundant SNPs, genotyped animals, or *Me* [40]. Investigating this dimensionality can help determine the sample size required for the discovery and training sets. Since the performance of GWA and genomic prediction depends on several factors, such as the genetic architecture of the trait, population structure, heritability, and sample size, more research is needed with real data to validate our findings.

## Conclusions

Accurate identification of causative variants from sequence data depends on the effective population size and, therefore, on the dimensionality of genomic information. Based on this dimensionality, the expected number of independent chromosome segments contains the additive genetic variance in a population. Consequently, using a GWA sample size larger than the expected number of independent chromosome segments has a limited impact on improving the resolution of the GWA and the identification of QTN. This is particularly relevant for populations with larger effective sizes, where the detection of QTN may be more effective. Therefore, the dimensionality of genomic information can provide valuable insights into selecting the suitable sample size for GWA and aid in identifying the most informative variants. Assigning genotyped animals with high reliability breeding values to the GWA discovery set helps better identify the significant QTN. As sequence data become available, preselecting variants and adding them to the regular chip data could improve prediction accuracy when the dimensionality of the genomic information is considered; however, the improvement is primarily limited.

## Supplementary Information

**Additional file 1.** QMSim parameter files for simul_Ne20_200QTL_h2_0.3.

**Additional file 2.** QMSim parameter files for simul_Ne20_200QTL_h2_0.9.

**Additional file 3.** QMSim parameter files for simul_Ne20_200QTL_h2_0.99.

**Additional file 4.** QMSim parameter files for simul_Ne20_2000QTL_h2_0.3.

**Additional file 5.** QMSim parameter files for simul_Ne20_2000QTL_h2_0.9.

**Additional file 6.** QMSim parameter files for simul_Ne20_2000QTL_h2_0.99.

**Additional file 7.** QMSim parameter files for simul_Ne200_200QTL_h2_0.3.

**Additional file 8.** QMSim parameter files for simul_Ne200_200QTL_h2_0.9.

**Additional file 9.** QMSim parameter files for simul_Ne200_200QTL_h2_0.99.

**Additional file 10.** QMSim parameter files for simul_Ne200_2000QTL_h2_0.3.

**Additional file 11.** QMSim parameter files for simul_Ne200_2000QTL_h2_0.9.

**Additional file 12.** QMSim parameter files for simul_Ne200_2000QTL_h2_0.99.

**Additional file 13: Figure S1.** a–l GWAS results for each scenario from one replicate: (a) *Ne*20 Q200 H30; (b) *Ne*20 Q200 H90; (c) *Ne*20 Q200 H99; (d) *Ne*20 Q2000 H30; (e) *Ne*20 Q2000 H90; (f) Ne20 Q2000 H99; (g) *Ne*200 Q200 H30; (h) *Ne*200 Q200 H90; (i) *Ne*200 Q200 H99; (j) *Ne*200 Q2000 H30; (k) *Ne*200 Q2000 H90; and (l) *Ne*200 Q2000 H99.

**Additional file 14: Tables S1.** a–d Number of significantly identified QTN, SNPs and the variance explained by QTN (mean ± SE) of five replicates: (a) Ne20Q200, (b) Ne20Q2000, (c) Ne200Q200, and (d) Ne200Q2000. **Tables S2.** a–d Estimated sample size using local polynomial regression $\left( {{\text{Sample}}_{{{\text{app}}1}} } \right)$ and for each feasible scenario: (a) Ne20 Q200, (b) Ne20 Q2000, (c) Ne200 Q200, and (d) Ne200 Q2000. %Var1: percentage of variance explained by significantly identified QTN; ${\text{SS}}_{{{\text{pol}}}}$2: estimated sample size using local polynomial regression; ${\text{EIGx}}_{{{\text{app}}1}}$3: EIGx scenario range including ${\text{Sample}}_{{{\text{app}}1}}$.

**Additional file 15: Table S3.** This table contains 5 sheets with the prediction accuracy and b1 values of each scenario Ne20 Q200, Ne20 Q2000, Ne200 Q200, and Ne200 Q2000, and PBLUP.

## Data Availability

The datasets used and/or analysed during the current study are available from the corresponding author on reasonable request.
