# Supplementary material for: Dimensionality of genomic information and its impact on genome-wide associations and variant selection for genomic prediction: a simulation study
Source: Genet Sel Evol. 2023 Jul 17;55:49. doi: 10.1186/s12711-023-00823-0 (PMC10351171; doi:10.1186/s12711-023-00823-0)
Supplement: Supplementary file 13 — Additional file 13: Figure S1. a–l GWAS results for each scenario from one replicate: (a) Ne20 Q200 H30; (b) Ne20 Q200 H90; (c) Ne20 Q200 H99; (d) Ne20 Q2000 H30; (e) Ne20 Q2000 H90; (f) Ne20 Q2000 H99; (g) Ne200 Q200 H30; (h) Ne200 Q200 H90; (i) Ne200 Q200 H99; (j) Ne200 Q2000 H30; (k) Ne200 Q2000 H90; and (l) Ne200 Q2000 H99. [file 12711_2023_823_MOESM13_ESM.docx]

**Additional file 13 Figure S1a to l**

**Figure S1 GWAS results for all scenarios from one replicate**

**(a)** **Ne20 Q200 H30**

| 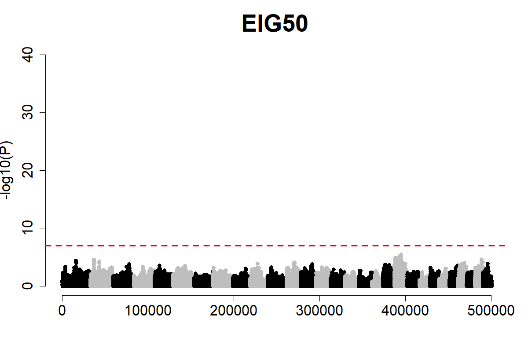 | 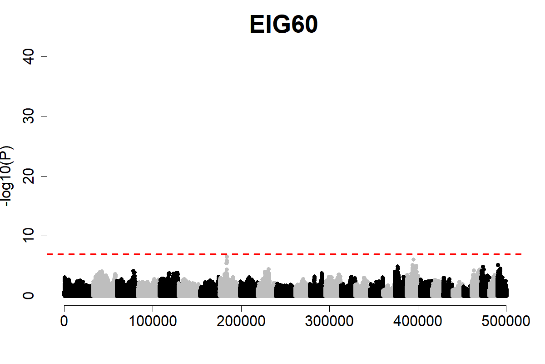 | 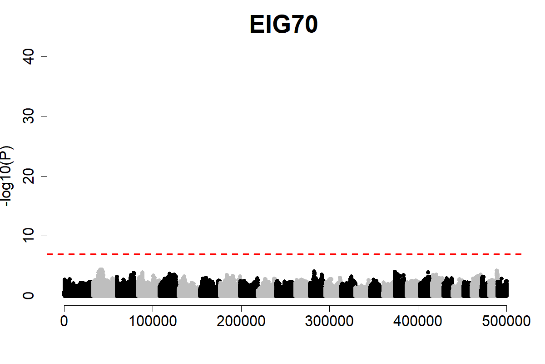 |
| --- | --- | --- |
| 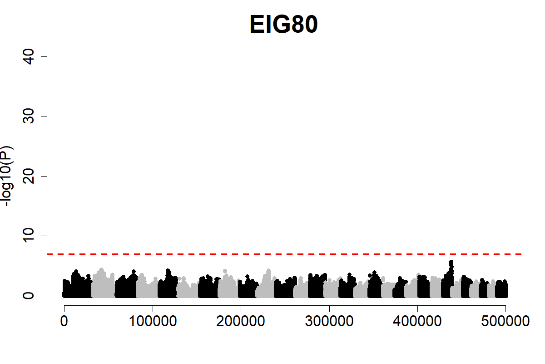 | 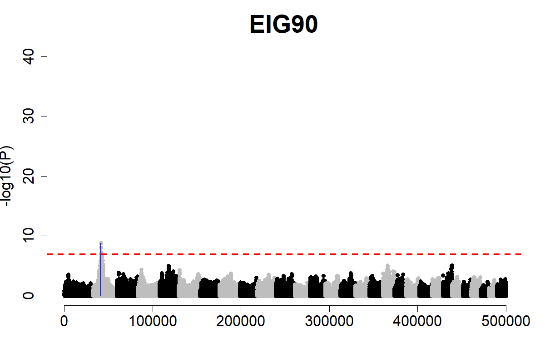 | 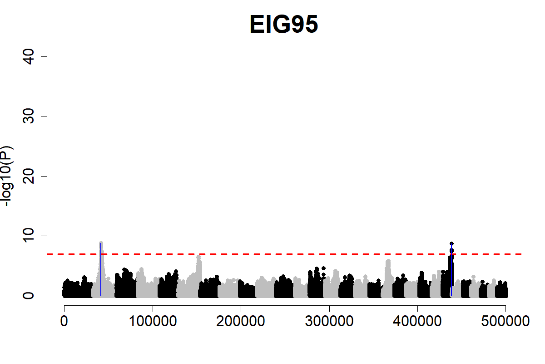 |
| 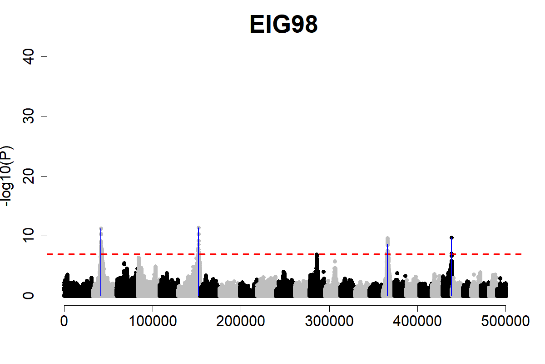 | 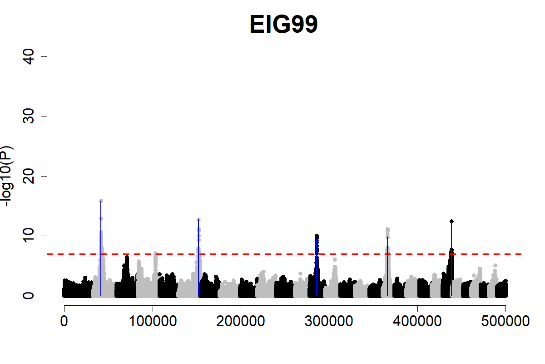 | 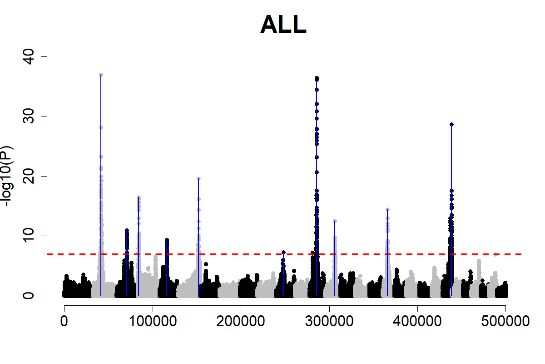 |

**(b)** **Ne20 Q200 H90**

| 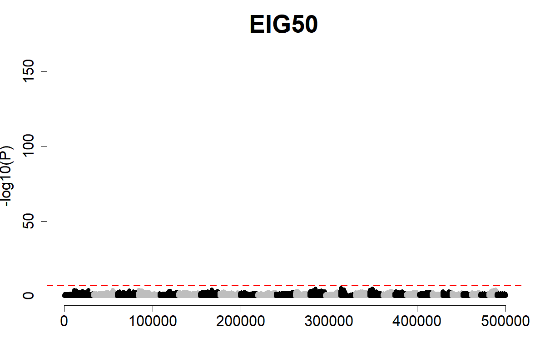 | 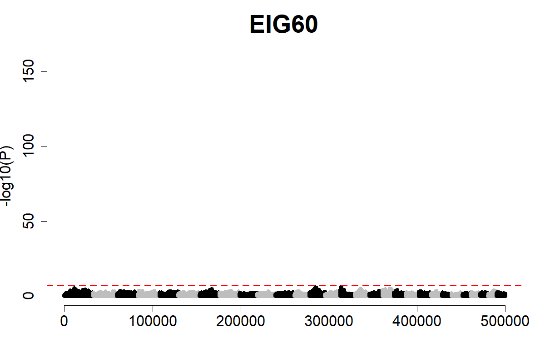 | 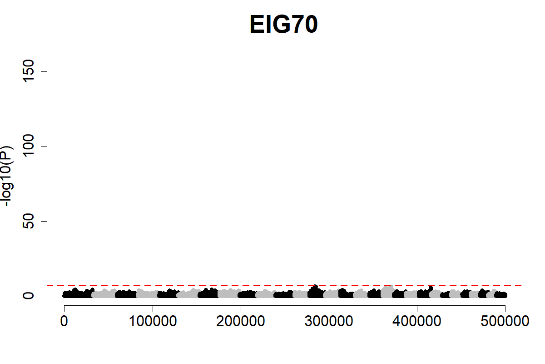 |
| --- | --- | --- |
| 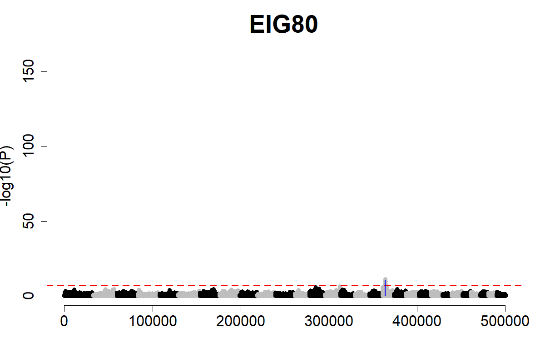 | 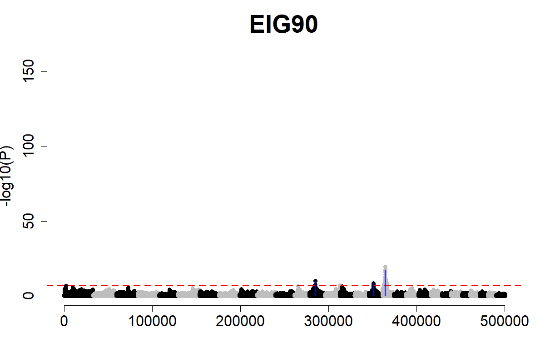 | 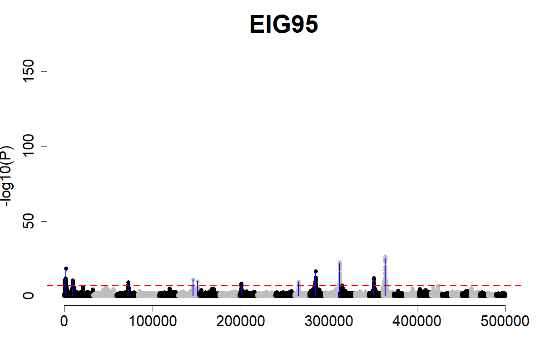 |
| 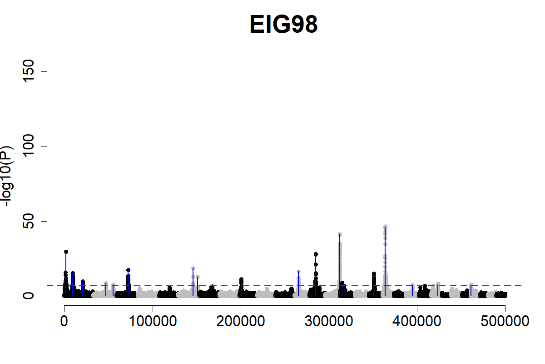 | 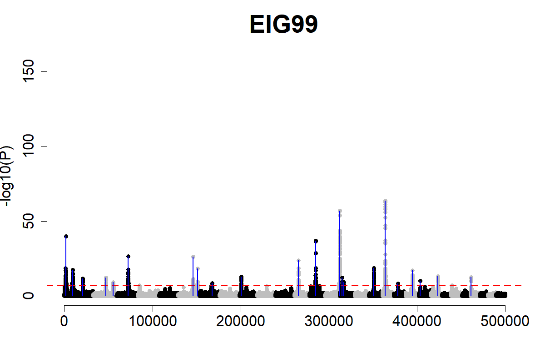 | 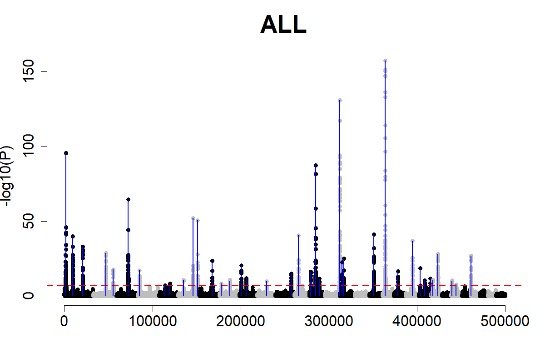 |

**(c)** **Ne20 Q200 H99**

| 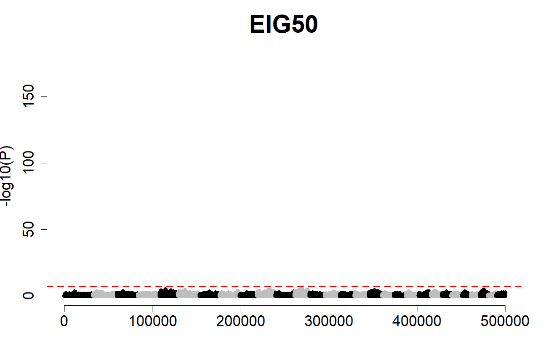 | 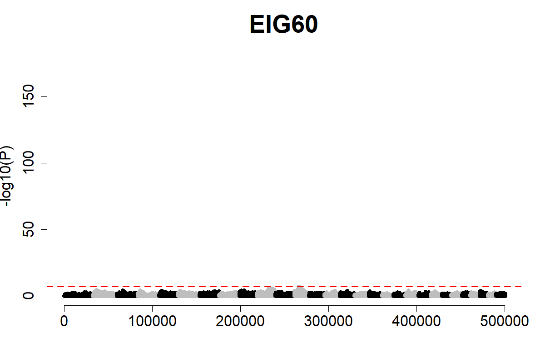 | 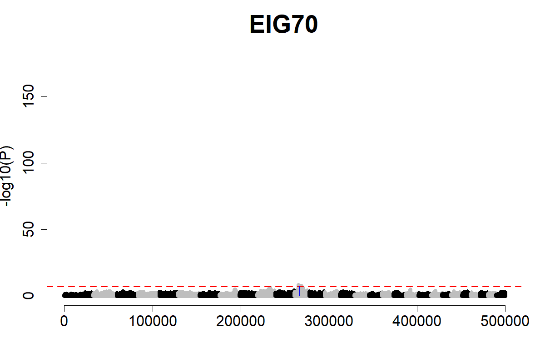 |
| --- | --- | --- |
| 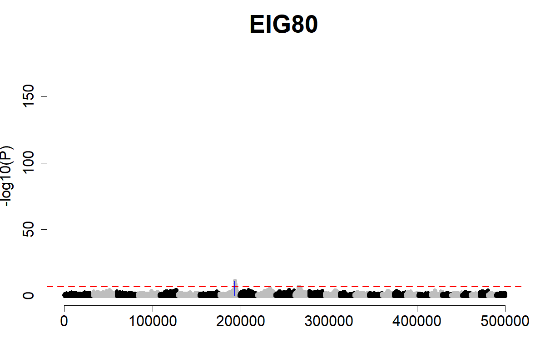 | 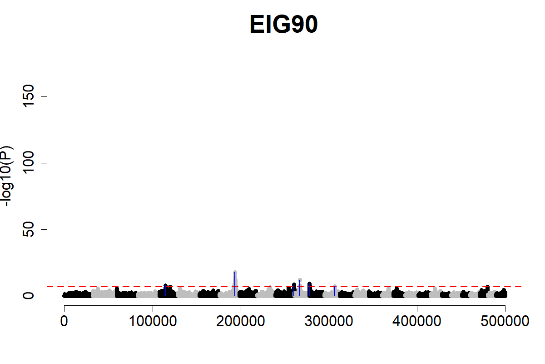 | 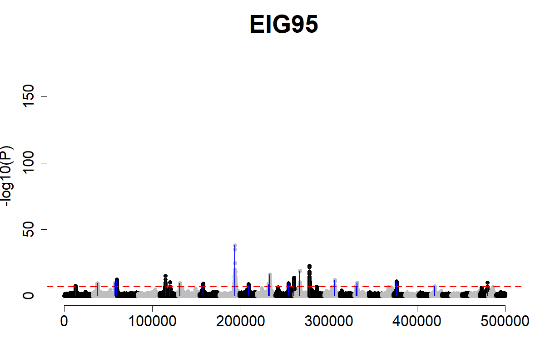 |
| 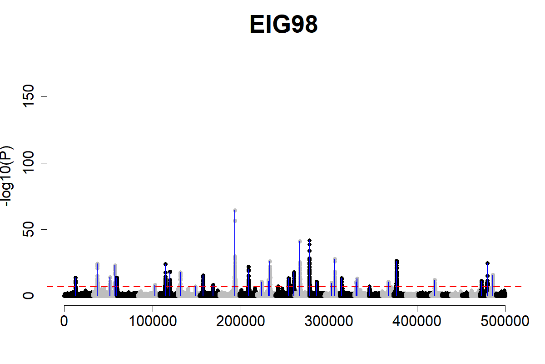 | 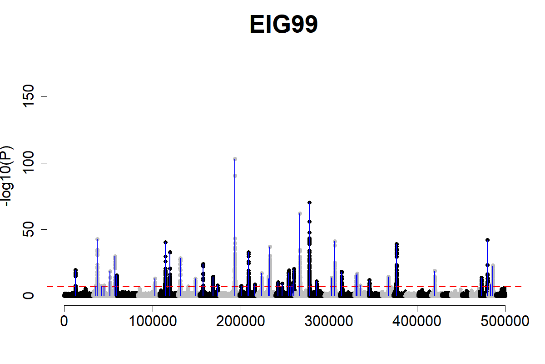 | 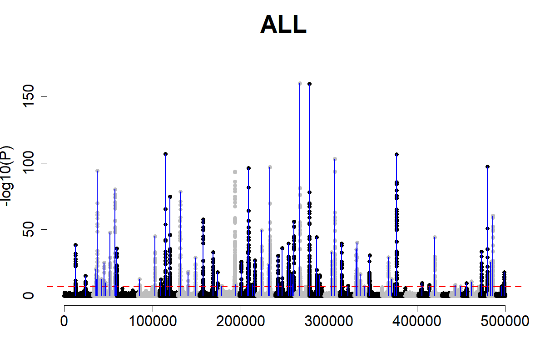 |

**(d)** **Ne20 Q2000 H30**

| 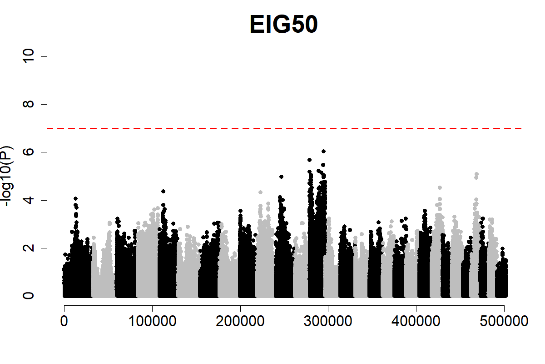 | 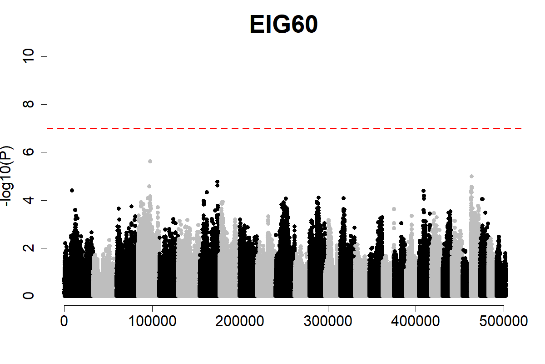 | 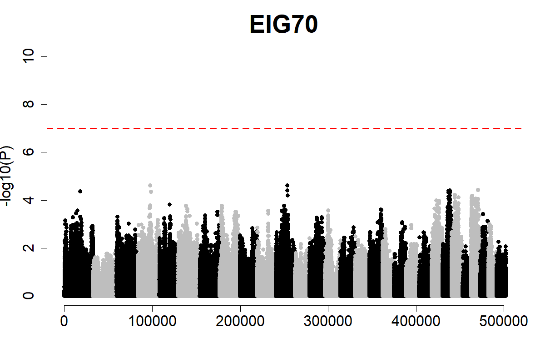 |
| --- | --- | --- |
| 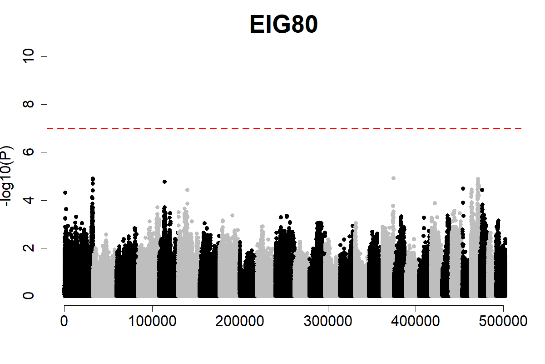 | 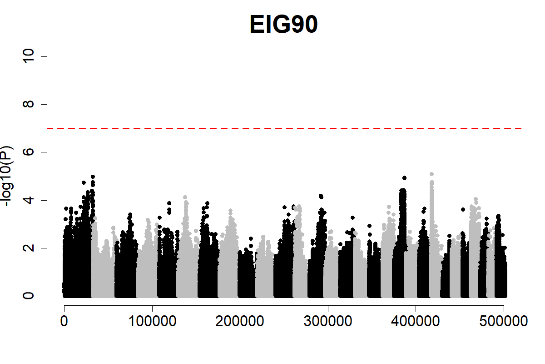 | 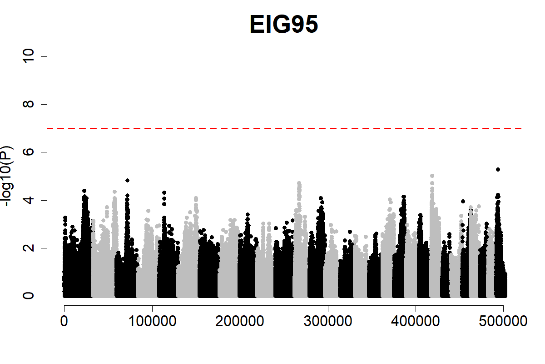 |
| 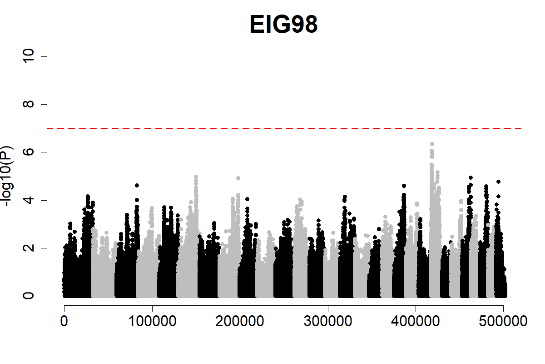 | 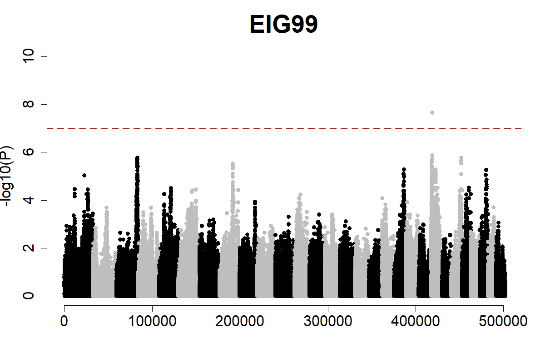 | 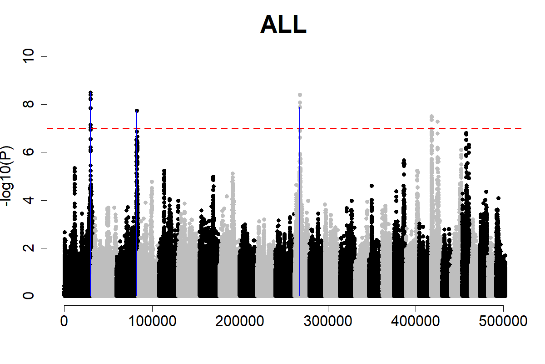 |

**(e)** **Ne20 Q2000 H90**

| 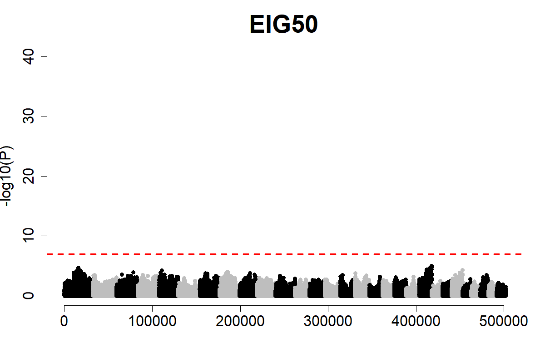 | 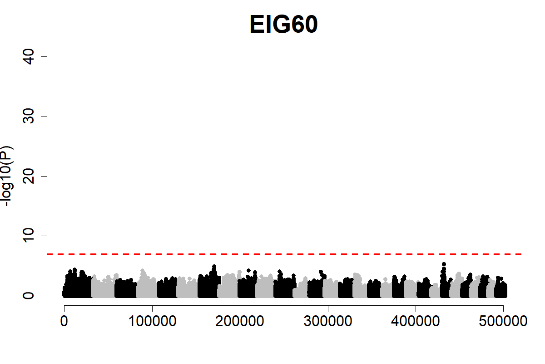 | 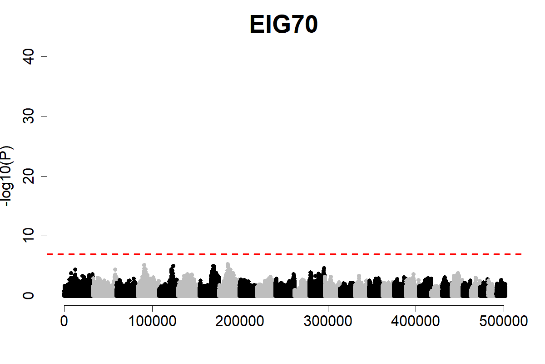 |
| --- | --- | --- |
| 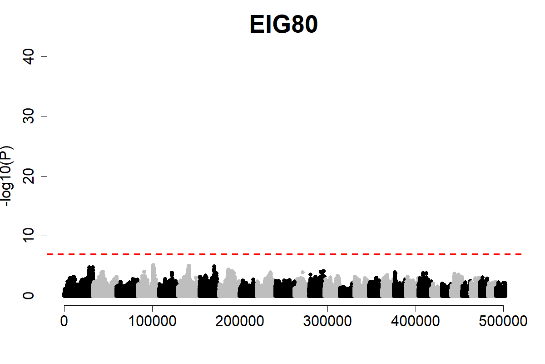 | 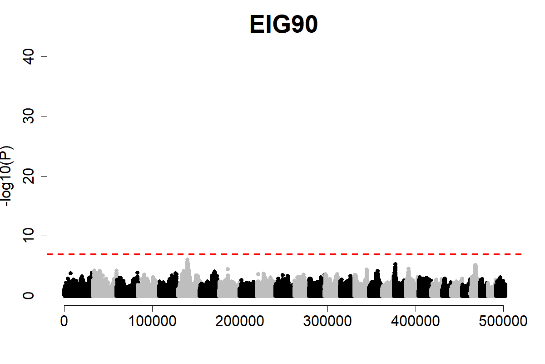 | 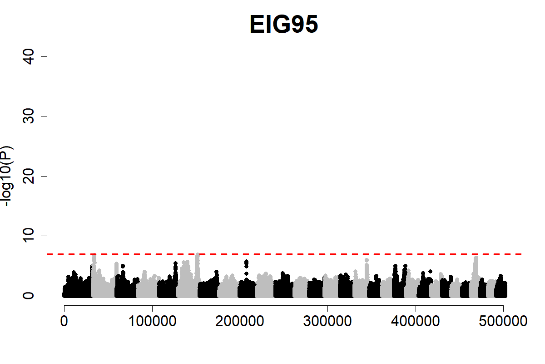 |
| 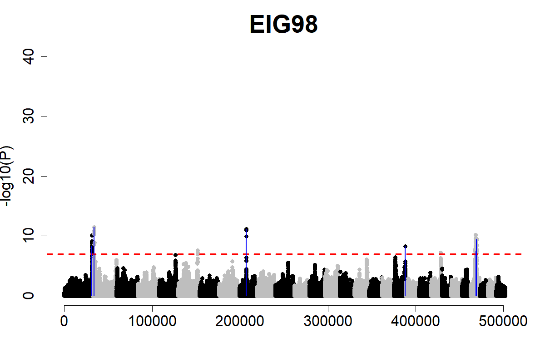 | 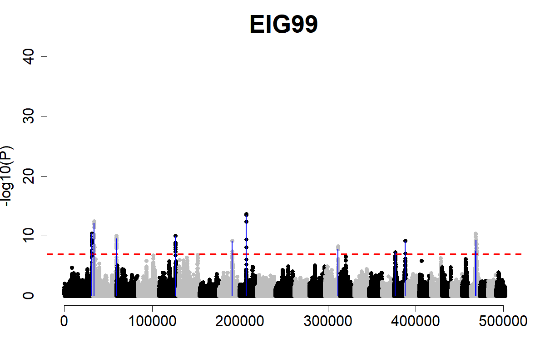 | 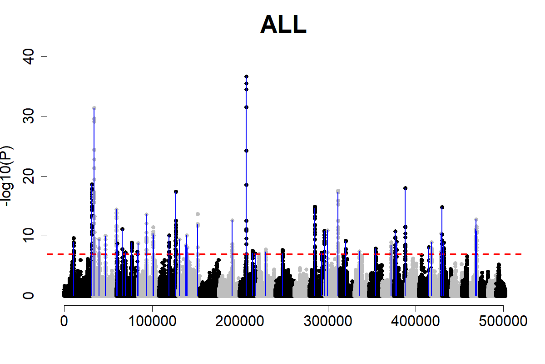 |

**(f)** **Ne20 Q2000 H99**

| 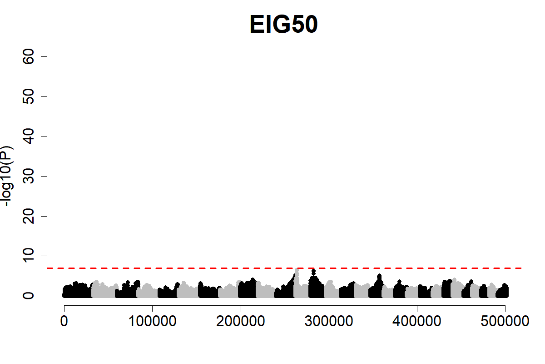 | 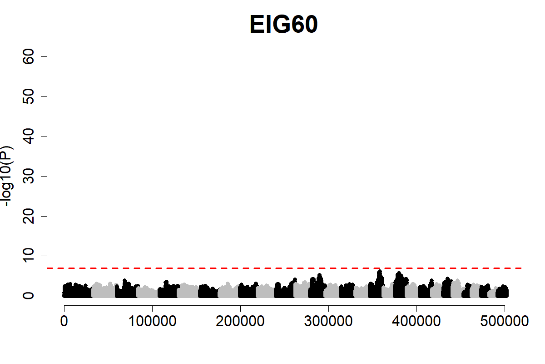 | 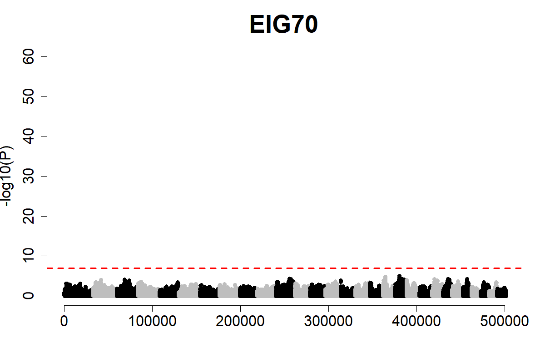 |
| --- | --- | --- |
| 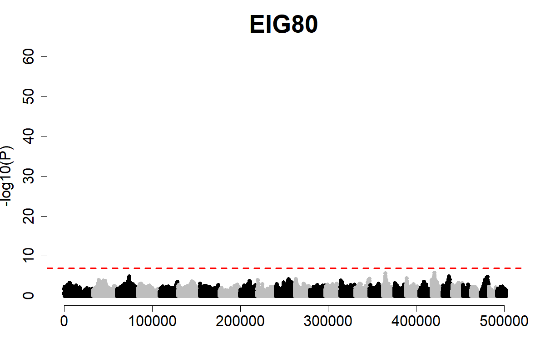 | 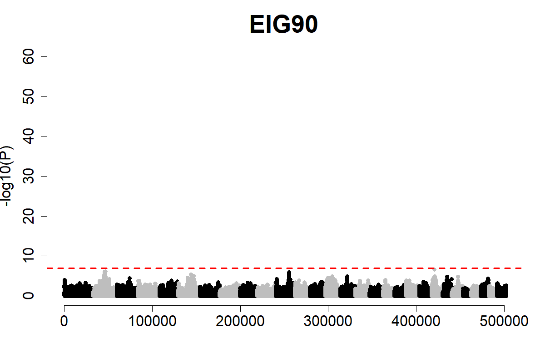 | 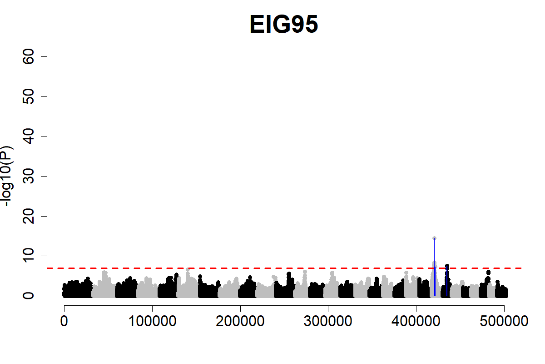 |
| 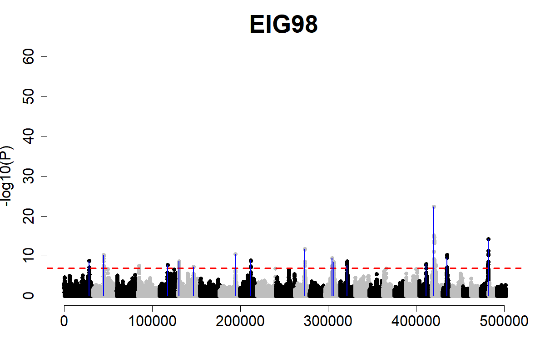 | 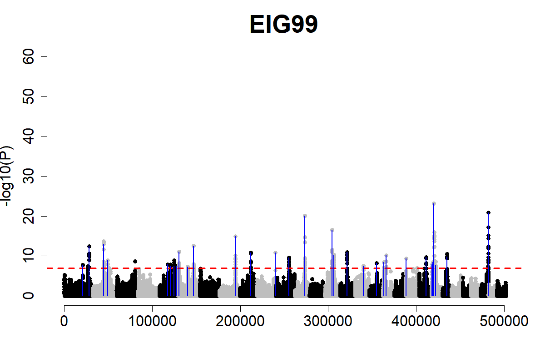 | 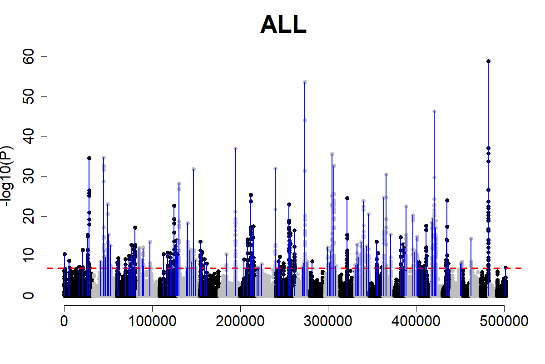 |

**(g)** **Ne200 Q200 H30**

| 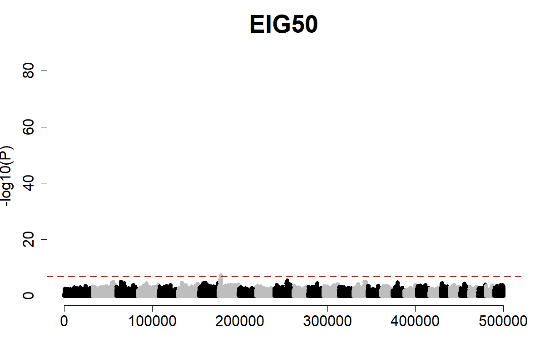 | 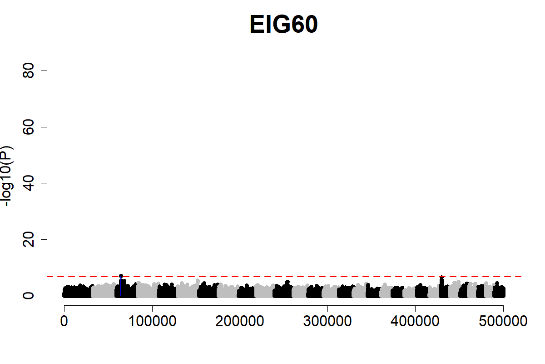 | 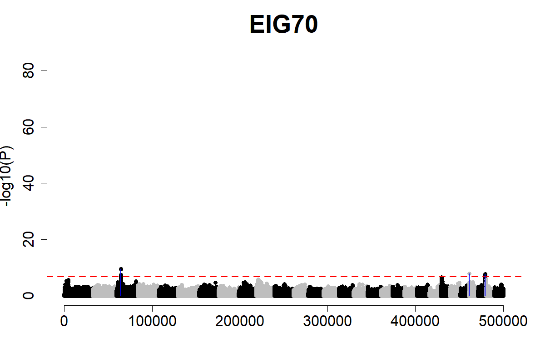 |
| --- | --- | --- |
| 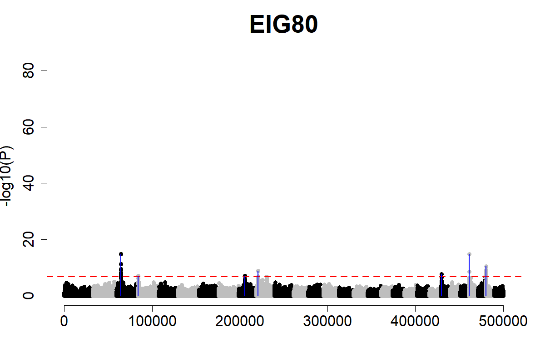 | 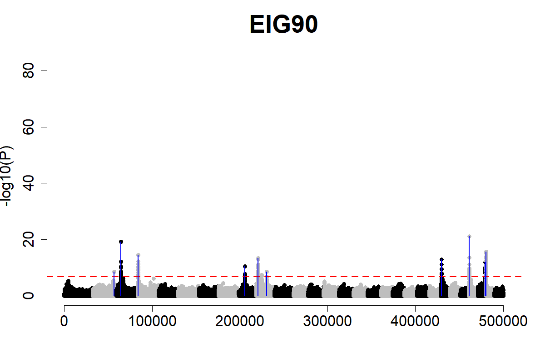 | 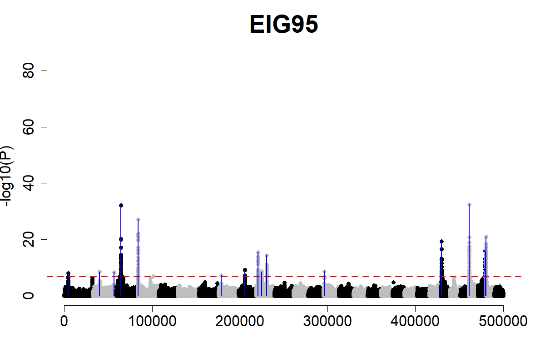 |
| 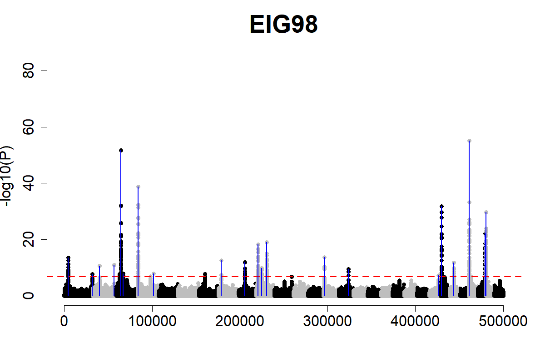 | 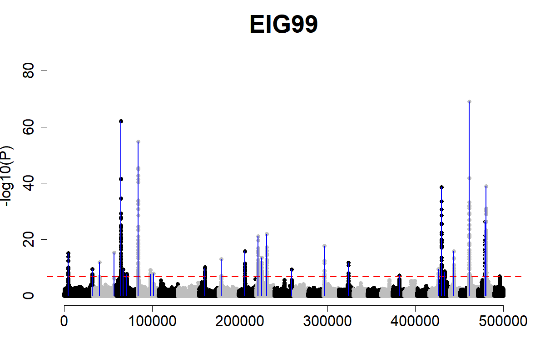 | 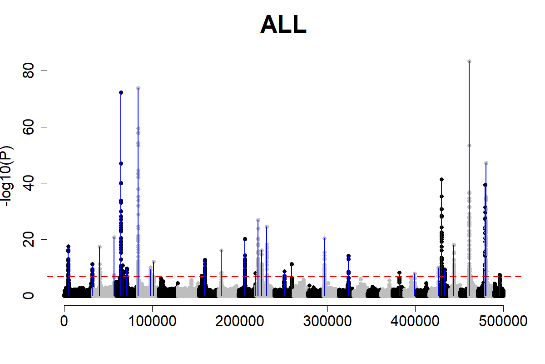 |

**(h)** **Ne200 Q200 H90**

| 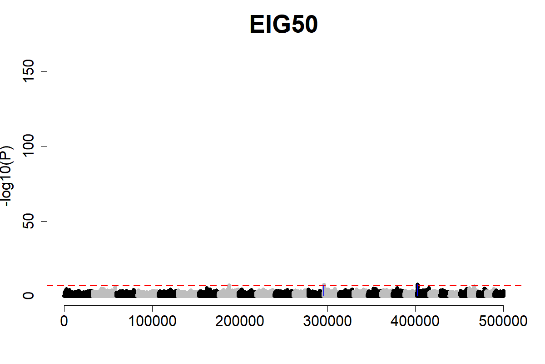 | 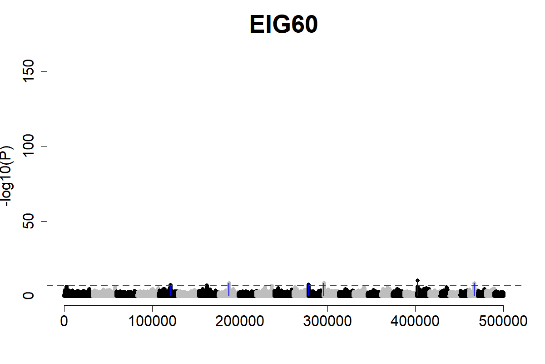 | 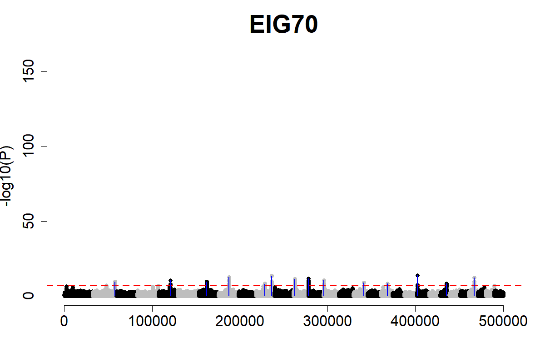 |
| --- | --- | --- |
| 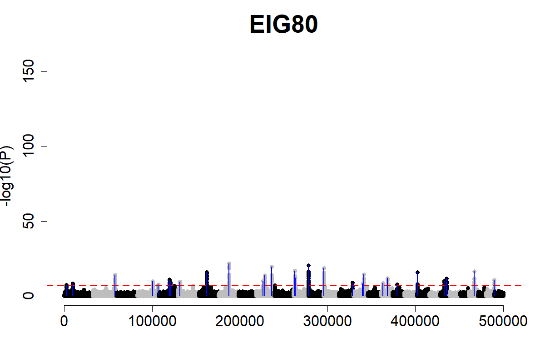 | 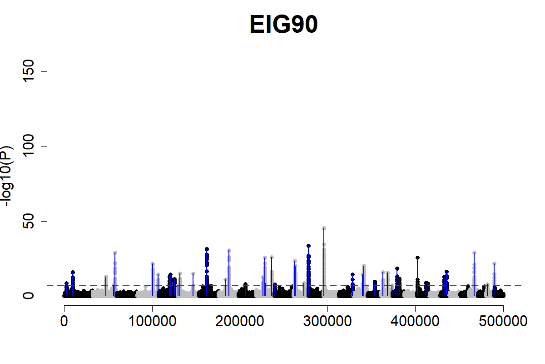 | 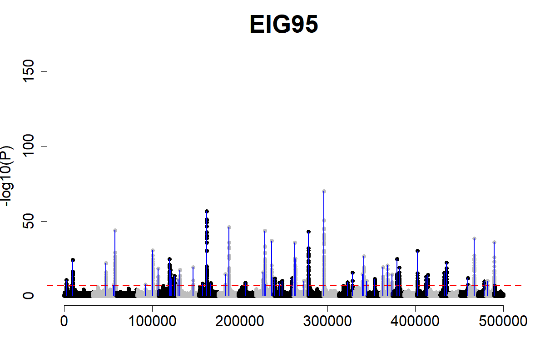 |
| 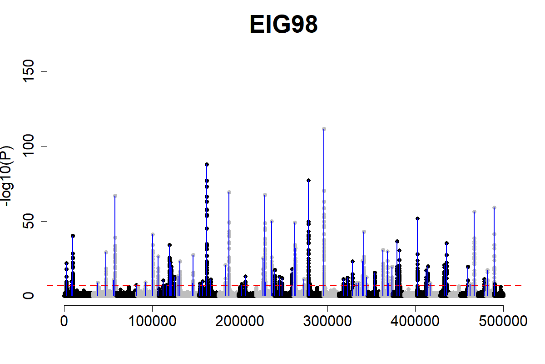 | 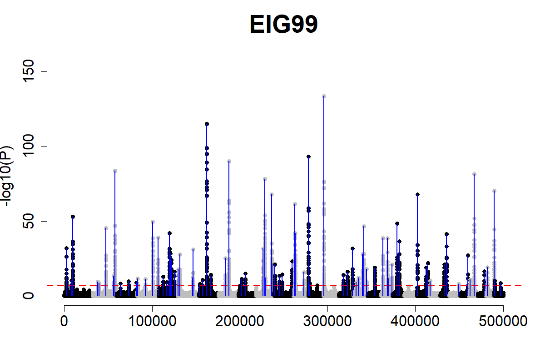 | 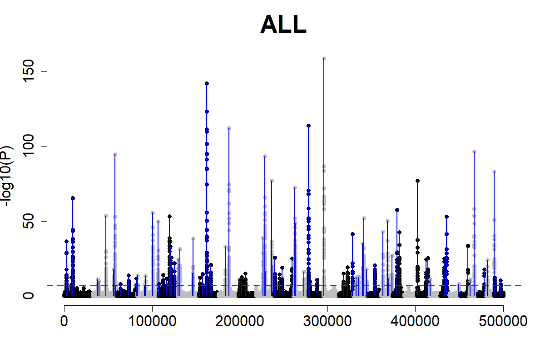 |

**(i)** **Ne200 Q200 H99**

| 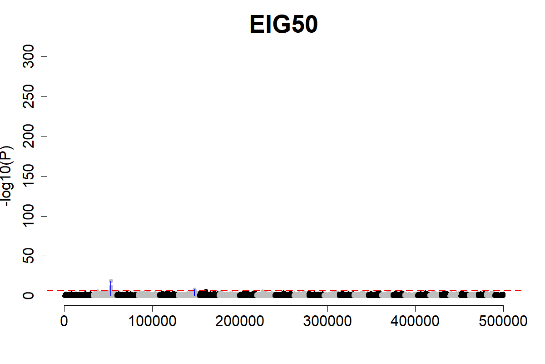 | 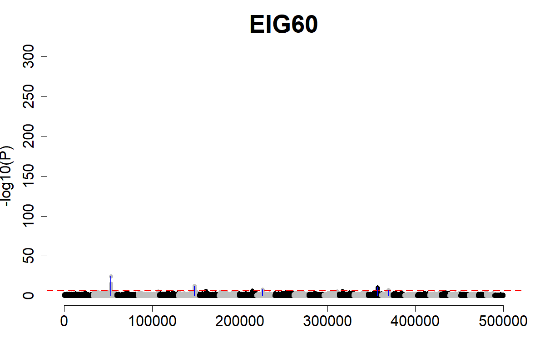 | 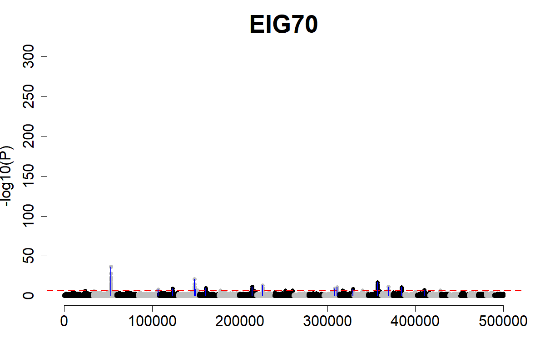 |
| --- | --- | --- |
| 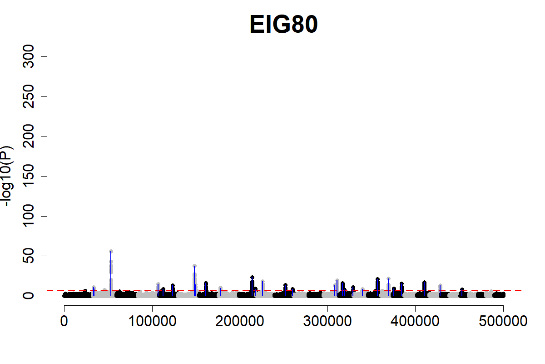 | 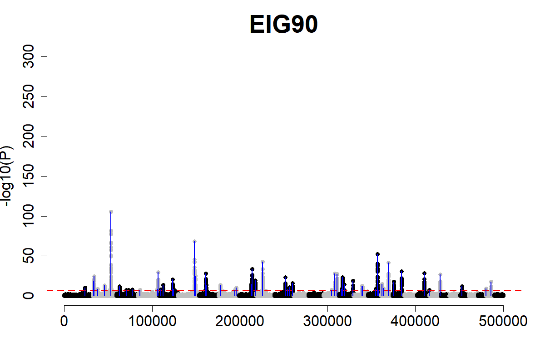 | 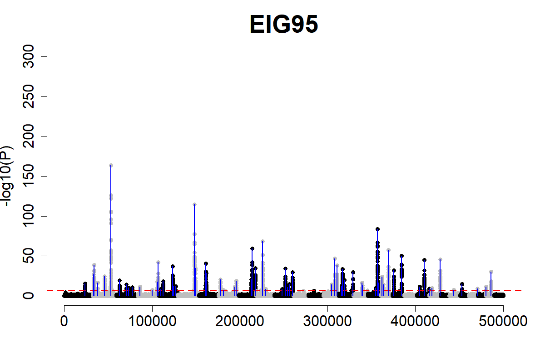 |
| 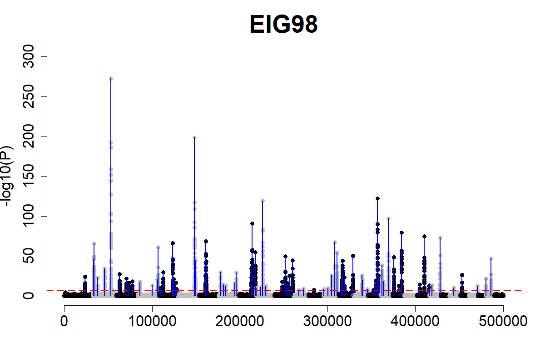 | 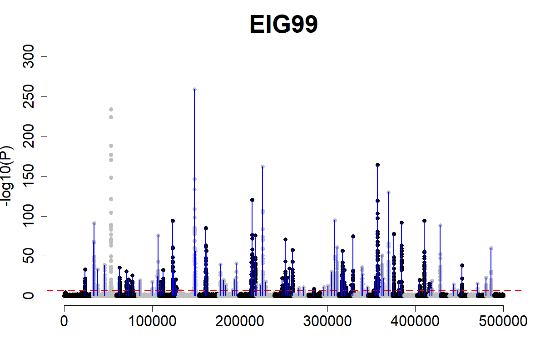 | 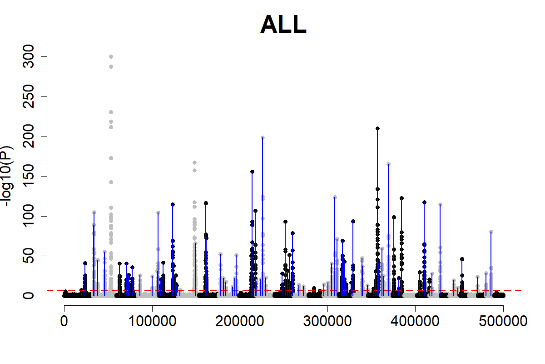 |

**(j)** **Ne200 Q2000 H30**

| 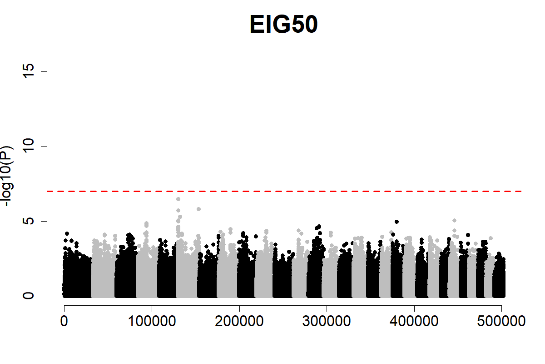 | 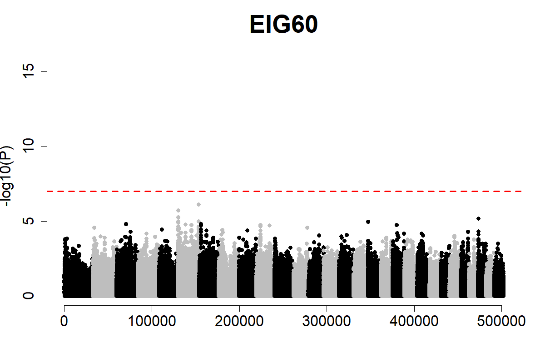 | 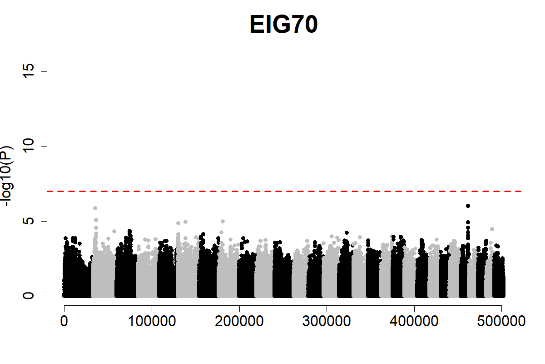 |
| --- | --- | --- |
| 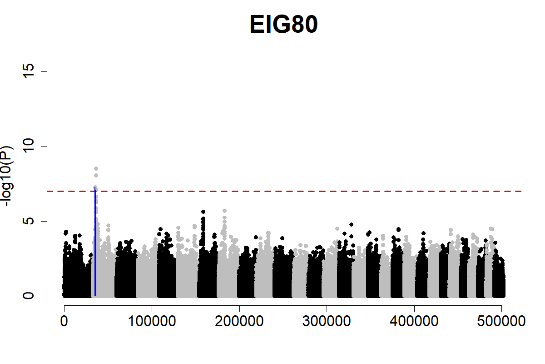 | 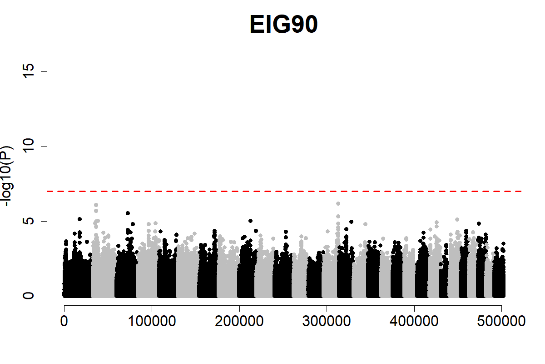 | 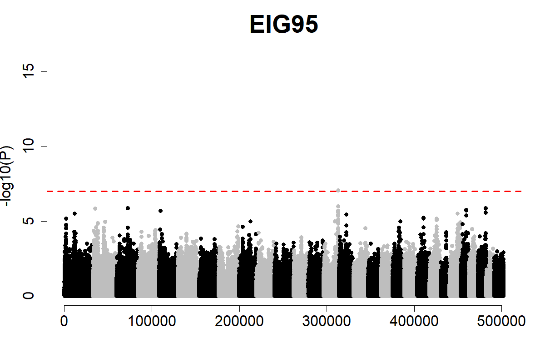 |
| 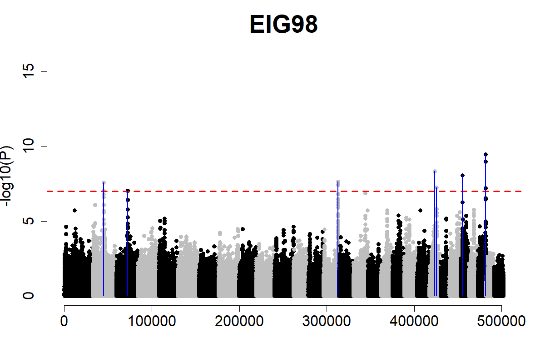 | 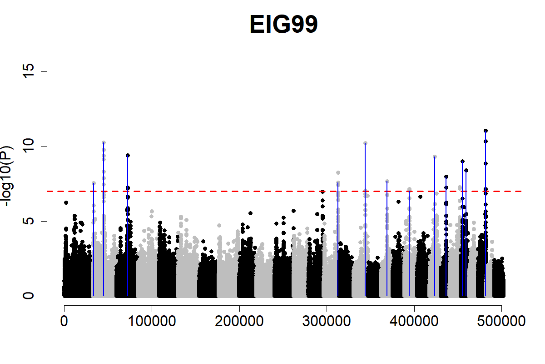 | 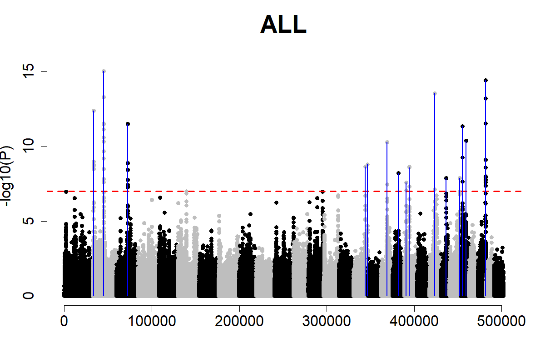 |

**(k)** **Ne200 Q2000 H90**

| 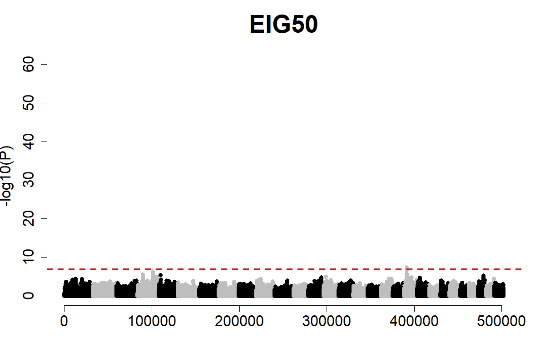 | 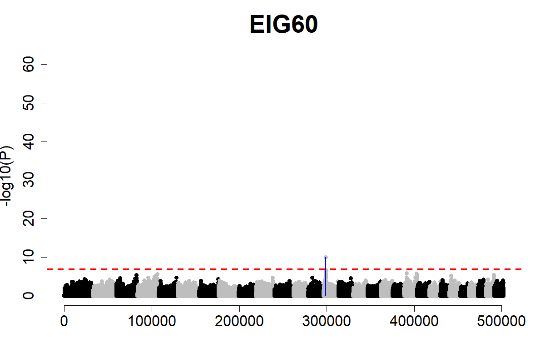 | 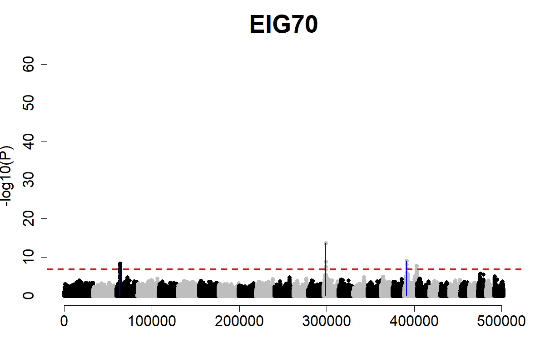 |
| --- | --- | --- |
| 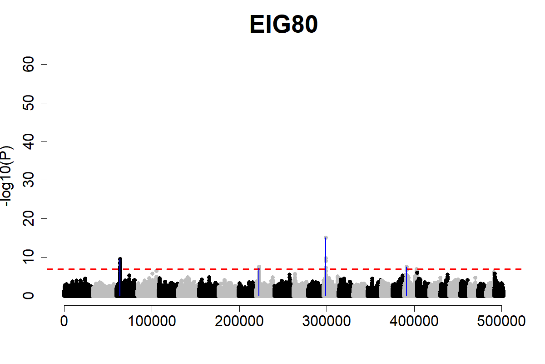 | 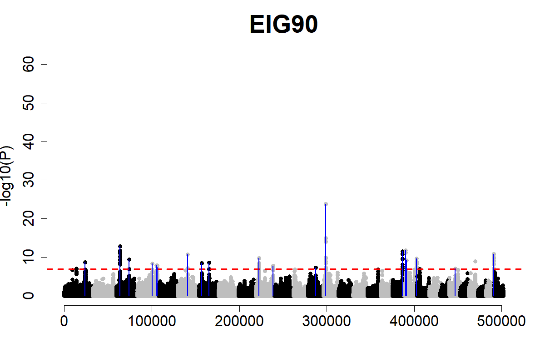 | 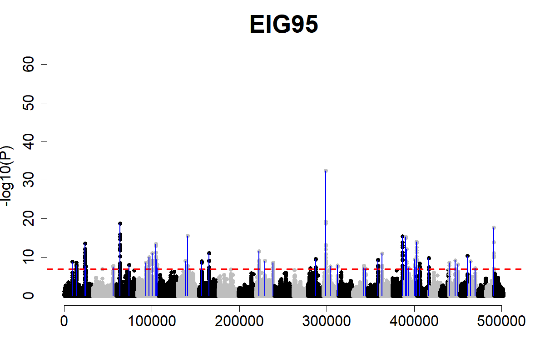 |
| 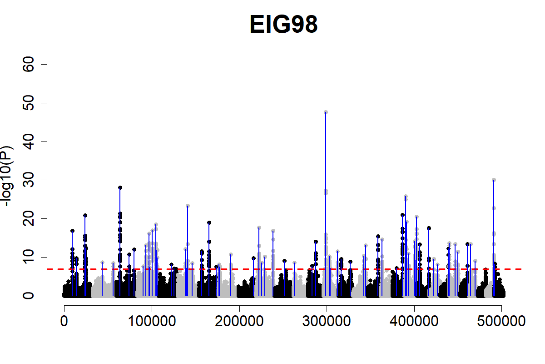 | 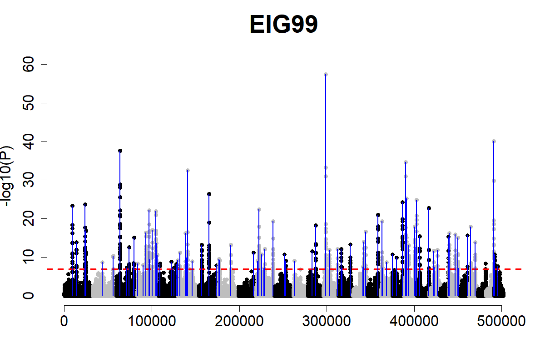 | 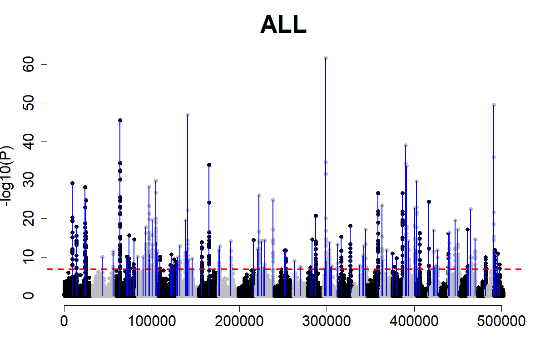 |

**(l)** **Ne200 Q2000 H99**

| 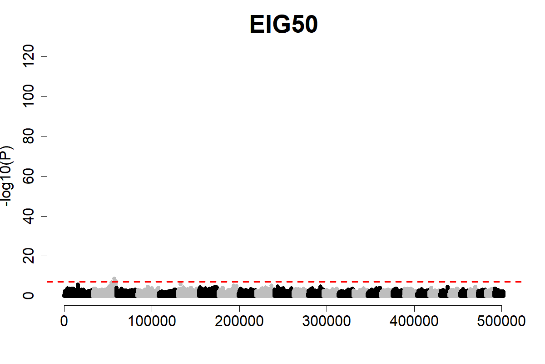 |  |  |
| --- | --- | --- |
|  |  |  |
|  |  |  |
