# Supplementary material for: Dimensionality of genomic information and its impact on genome-wide associations and variant selection for genomic prediction: a simulation study
Source: Genet Sel Evol. 2023 Jul 17;55:49. doi: 10.1186/s12711-023-00823-0 (PMC10351171; doi:10.1186/s12711-023-00823-0)
Supplement: Supplementary file 14 — Additional file 14: Tables S1. a–d Number of significantly identified QTN, SNPs and the variance explained by QTN (mean ± SE) of five replicates: (a) Ne20Q200, (b) Ne20Q2000, (c) Ne200Q200, and (d) Ne200Q2000. Tables S2. a–d Estimated sample size using local polynomial regression $\left( {{\text{Sample}}_{{{\text{app}}1}} } \right)$Sampleapp1 and for each feasible scenario: (a) Ne20 Q200, (b) Ne20 Q2000, (c) Ne200 Q200, and (d) Ne200 Q2000. %Var1: percentage of variance explained by significantly identified QTN; ${\text{SS}}_{{{\text{pol}}}}$SSpol2: estimated sample size using local polynomial regression; ${\text{EIGx}}_{{{\text{app}}1}}$EIGxapp13: EIGx scenario range including ${\text{Sample}}_{{{\text{app}}1}}$Sampleapp1. [file 12711_2023_823_MOESM14_ESM.docx]

**Additional file 14 Tables S1a, b, c, and d**

**Number of significantly identified QTN, SNPs and the variance explained by QTN (mean ± SE)** **of five replicates**

|  | H30 | | H90 | | H99 | |
| --- | --- | --- | --- | --- | --- | --- |
|  | NQTN | NQTN +SNP | NQTN | NQTN + SNP | NQTN | NQTN + SNP |
| EIG50 | 0 (0) | 0 | 0 (0) | 0 | 0 (0) | 2.0±1.3 |
| EIG60 | 0 (0) | 0 | 0 (0) | 0 | 0.4±0.4 (4.6±4.6) | 10.4±8.1 |
| EIG70 | 0 (0) | 0 | 0.2±0.2 (2.9±2.9) | 10.6±10.6 | 1.4±0.4 (9.4±3.9) | 64.4±34.6 |
| EIG80 | 0 (0) | 0 | 1.0±0.3 (9.2±2.6) | 51.0±36.3 | 2.2±0.5 (16.1±2.9) | 210.8±91.0 |
| EIG90 | 0.6±0.2 (7.0±3.8) | 5.0±1.9 | 4.6±0.8(22.4±3.0) | 126.8±52.3 | 8.2±1.1 (33.9±3.8) | 361.8±91.6 |
| EIG95 | 1.0±0.3 (11.4±3.4) | 13.4±4.5 | 9.6±1.6 (36.1±2.9) | 273.2±64.4 | 19.0±1.5 (52.1±2.4) | 659.4±67.4 |
| EIG98 | 3.2±0.4 (24.9±3.0) | 113.6±24.7 | 19.8±1.1 (52.2±2.1) | 546.8±58.3 | 32.2±2.0 (65.2±2.0) | 1084.2±27.0 |
| EIG99 | 4.6±0.2 (31.0±2.9) | 229.4±48.8 | 26.2±1.6 (60.0±1.6) | 672.2±90.6 | 42.2±2.4 (71.2±2.0) | 1324.0±18.4 |
| All | 12.6±1.2 (50.1±2.0) | 642.6±92.8 | 43.8±1.6 (70.7±1.7) | 1235.4±16.9 | 67.0±2.4 (77.6±1.7) | 1540.2±23.4 |

**(a) Ne20Q200**

**(b) Ne20Q2000**

|  | H30 | | H90 | | H99 | |
| --- | --- | --- | --- | --- | --- | --- |
|  | NQTN | NQTN +SNP | NQTN | NQTN+SNP | NQTN | NQTN+SNP |
| EIG50 | 0 (0) | 0 | 0 (0) | 0 | 0 (0) | 0 |
| EIG60 | 0 (0) | 0 | 0 (0) | 0 | 0 (0) | 0 |
| EIG70 | 0 (0) | 0 | 0 (0) | 0 | 0 (0) | 0 |
| EIG80 | 0 (0) | 0 | 0 (0) | 0.2±0.2 | 0 (0) | 3.2±3.2 |
| EIG90 | 0.2±0.2 (0.3±0.3) | 1.2±1.2 | 0.2±0.2 (0.2±0.2) | 1.4±1.4 | 0 (0) | 0.4±0.4 |
| EIG95 | 0.2±0.2 (0.3±0.3) | 7.0±7.0 | 0.2±0.2 (0.3±0.3) | 2.0±1.5 | 3.6±0.4 (3.7±0.7) | 20.2±2.9 |
| EIG98 | 0.2±0.2 (0.3±0.3) | 2.8±2.6 | 4.4±1.5 (3.9±1.3) | 36.6±12.9 | 14.8±1.4 (11.3±1.0) | 112.2±18.6 |
| EIG99 | 0.4±0.4 (0.5±0.5) | 5.0±4.8 | 10.4±1.7 (8.2±1.7) | 74.4±19.3 | 34.8±2.3 (20.8±1.8) | 253.6±34.5 |
| All | 2.8±0.7 (3.5±0.6) | 26.4±10.8 | 46.0±1.5 (24.3±1.4) | 344.2±39.2 | 138.4±5.7 (45.0±1.1) | 922.8±43.6 |

|  | H30 | | H90 | | H99 | |
| --- | --- | --- | --- | --- | --- | --- |
|  | NQTN | NQTN+SNP | NQTN | NQTN+SNP | NQTN | NQTN+SNP |
| EIG50 | 0 (0) | 0.4±0.2 | 4.0±1.0(26.8±5.9) | 26.2±6.6 | 2.8±0.7(26.8±3.3) | 26.4±4.7 |
| EIG60 | 0.6±0.2(7.2±3.5) | 4.8±3.8 | 7.2±0.7(37.7±5.5) | 66.0±14.8 | 6.8±1.3(39.4±4.2) | 58.6±9.0 |
| EIG70 | 2.0±0.4(16.6±4.0) | 9.4±4.8 | 12.6±1.1(52.1±4.4) | 141.8±16.2 | 14.0±1.1(55.2±3.0) | 155.8±12.7 |
| EIG80 | 4.0±0.8(27.9±3.7) | 26.2±6.6 | 21.6±2.1(66.0±1.8) | 278.4±13.4 | 24.6±1.2(69.9±1.3) | 322.8±9.7 |
| EIG90 | 9.4±0.6(45.7±1.2) | 88.4±8.6 | 33.6±4.5(76.5±1.5) | 504.2±10.6 | 43.0±3.3(84.1±1.3) | 664.8±21.5 |
| EIG95 | 13.6±1.3(54.1±2.7) | 174.4±12.8 | 46.0±5.2(84.3±1.8) | 712.2±17.4 | 55.4±3.1(89.6±1.2) | 924.6±28.9 |
| EIG98 | 21.2±1.1(64.6±2.8) | 281.8±18.1 | 56.8±6.0(88.2±1.7) | 929.6±23.5 | 68.4±3.2(93.2±1.1) | 1139.4±27.2 |
| EIG99 | 27.6±1.4(70.7±2.1) | 360.6±20.5 | 63.4±5.6(90.0±1.5) | 1055.2±32.4 | 74.6±2.3(94.4±1.0) | 1227.4±30.3 |
| All | 32.4±1.2(74.5±1.0) | 451.0±21.9 | 69.0±5.7(91.5±1.3) | 1149.8±29.5 | 82.0±1.9(95.5±1.1) | 1273.4±22.7 |

**(c) Ne200Q200**

**(d) Ne200Q2000**

|  | H30 | | H90 | | H99 | |
| --- | --- | --- | --- | --- | --- | --- |
|  | NQTN | NQTN+SNP | NQTN | NQTN+SNP | NQTN | NQTN+SNP |
| EIG50 | 0 (0) | 0 | 0 (0) | 0.2±0.2 | 0 (0) | 1.2±0.7 |
| EIG60 | 0 (0) | 0.2±0.2 | 0.6±0.4(1.3±0.8) | 3.8±3.3 | 1.0±0.4(2.8±1.2) | 3.6±1.9 |
| EIG70 | 0 (0) | 0 | 1.6±0.5(2.5±0.8) | 7.6±3.7 | 3.6±0.5(6.8±1.0) | 16.0±3.9 |
| EIG80 | 0.2±0.2(0.1±0.1) | 1.6±1.0 | 5.6±0.8(7.5±1.4) | 27.0±7.9 | 10.6±0.7(14.1±1.4) | 58.6±5.1 |
| EIG90 | 1.0±0.3(1.7±0.9) | 1.8±0.7 | 22.4±1.2(20.5±1.0) | 106.0±14.9 | 33.2±1.7(27.0±1.8) | 190.4±15.0 |
| EIG95 | 1.8±0.9(2.9±1.5) | 6.6±3.2 | 43.0±1.5(30.8±0.7) | 231.8±15.0 | 70.6±3.5(40.8±1.2) | 413.6±21.1 |
| EIG98 | 6.6±1.2(8.3±1.8) | 25.0±7.4 | 70.8±2.2(40.5±0.8) | 427.6±20.9 | 126.8±3.5(54.3±1.0) | 728.8±15.6 |
| EIG99 | 11.2±1.2(11.9±1.7) | 48.0±12.5 | 93.6±3.0(46.6±0.2) | 557.8±16.4 | 170.6±5.4(61.6±0.6) | 931.6±15.5 |
| All | 18.0±1.3(17.0±1.7) | 88.8±15.9 | 117.2±3.4(51.5±0.7) | 691.0±13.9 | 217.6±6.7(67.6±0.8) | 1080.8±15.1 |

Values in the bracket are the %Variance explained by identified QTN

**Additional file 14 Tables S2a, b, c, and d**

**Estimated sample size using local polynomial regression (**$\mathbf{Sample}_{\mathbf{app1}}\boldsymbol{)}$ **and for all feasible scenarios**

**(a) Ne20 Q200**

| **Heritability** | **%Var^1^** | $\mathbf{SS}_{\mathbf{pol}}$**^2^** | $\mathbf{EIGx}_{\mathbf{app1}}$**^3^** |
| --- | --- | --- | --- |
| H30 (ALL) | 50.1 |  |  |
| H90 | 50.1 | 3626 | EIG95~98 (1719~3952) |
| H99 | 50.1 | 1622 | EIG90~95 (871~1728) |
| H30 (EIG99) | 31.0 |  |  |
| H90 | 31.0 | 1399 | EIG90~95 (867~1719) |
| H99 | 31.0 | 785 | EIG80~90 (395~871) |
| H30 (EIG98) | 24.9 |  |  |
| H90 | 24.9 | 1020 | EIG90~95 (867~1719) |
| H99 | 24.9 | 616 | EIG80~90 (395~871) |
| H30 (EIG95) | 11.4 |  |  |
| H90 | 11.4 | 486 | EIG80~90 (391~867) |
| H99 | 11.4 | 278 | EIG70~80 (220~395) |
| H30 (EIG90) | 7.0 |  |  |
| H90 | 7.0 | 388 | EIG70~80 (219~391) |
| H99 | 7.0 | 189 | EIG60~70 (130~220) |

**(b) Ne20 Q2000**

| **Heritability** | **%Var^1^** | $\mathbf{SS}_{\mathbf{pol}}$**^2^** | $\mathbf{EIGx}_{\mathbf{app1}}$**^3^** |
| --- | --- | --- | --- |
| H30 (ALL) | 3.5 |  |  |
| H90 | 3.5 | 3652 | EIG95~98 (1674~3854) |
| H99 | 3.5 | 1599 | EIG90~95 (833~1658) |

**(c) Ne200 Q200**

| **Heritability** | **%Var^1^** | $\mathbf{SS}_{\mathbf{pol}}$**^2^** | $\mathbf{EIGx}_{\mathbf{app1}}$**^3^** |
| --- | --- | --- | --- |
| H30 (ALL) | 74.5 |  |  |
| H90 | 74.5 | 4458 | EIG80~90 (2617~5182) |
| H99 | 74.5 | 3153 | EIG80~90 (2598~5146) |
| H30 (EIG99) | 70.7 |  |  |
| H90 | 70.7 | 3475 | EIG80~90 (2617~5182) |
| H99 | 70.7 | 2543 | EIG70~80 (1507~2598) |
| H0.3 (EIG98) | 64.6 |  |  |
| H0.9 | 64.6 | 2427 | EIG70~80 (1515~2617) |
| H0.99 | 64.6 | 2190 | EIG70~80 (1507~2598) |
| H30 (EIG95) | 54.1 |  |  |
| H90 | 54.1 | 1752 | EIG70~80 (1515~2617) |
| H99 | 54.1 | 1582 | EIG70~80 (1507~2598) |
| H30 (EIG90) | 45.7 |  |  |
| H90 | 45.7 | 1292 | EIG60~70 (900~1515) |
| H99 | 45.7 | 1202 | EIG60~70 (896~1507) |
| H30 (EIG80) | 27.9 |  |  |
| H90 | 27.9 | 516 | < EIG50 (< 517) |
| H99 | 27.9 | NA | NA |
| H30 (EIG70) | 16.6 |  |  |
| H90 | 16.6 | NA | NA |
| H99 | 16.6 | NA | NA |
| H30 (EIG60) | 7.2 |  |  |
| H90 | 7.2 | NA | NA |
| H99 | 7.2 | NA | NA |

**(d) Ne200 Q2000**

| **Heritability** | **%Var^a^** | $\mathbf{SS}_{\mathbf{pol}}$**^b^** | $\mathbf{EIGx}_{\mathbf{app1}}$**^c^** |
| --- | --- | --- | --- |
| H30 (ALL) | 17.0 |  |  |
| H90 | 17.0 | 4526 | EIG80~90 (2609~5175) |
| H99 | 17.0 | 3206 | EIG80~90 (2612~5172) |
| H30 (EIG99) | 11.9 |  |  |
| H90 | 11.9 | 3528 | EIG80~90 (2609~5175) |
| H99 | 11.9 | 2287 | EIG70~80 (1514~2612) |
| H30 (EIG98) | 8.3 |  |  |
| H90 | 8.3 | 2824 | EIG80~90 (2609~5175) |
| H99 | 8.3 | 1720 | EIG70~80 (1514~2612) |
| H30 (EIG95) | 2.9 |  |  |
| H90 | 2.9 | 1481 | EIG60~70 (895~1509) |
| H99 | 2.9 | 924 | EIG60~70 (900~1514) |
| H30 (EIG90) | 1.7 |  |  |
| H90 | 1.7 | 1104 | EIG60~70 (895~1509) |
| H99 | 1.7 | 757 | EIG50~EIG60 (518~900) |

^a^%Var: Percentage of variance explained by significantly identified QTN

^b^$\mathrm{SS}_{\mathrm{pol}}$: Estimated sample size using local polynomial regression

^c^$\mathrm{EIGx}_{app1}$: EIGx scenario range including $\mathrm{Sample}_{app1}$
